# Direct disassembly of α-syn preformed fibrils into α-syn monomers by an all-D-peptide

**DOI:** 10.1038/s41531-025-01132-7

**Published:** 2025-09-22

**Authors:** Marc Sevenich, Ian Gering, Bettina Kass, Madita Vollmer, Selma Aghabashlou Saisan, Markus Tusche, Tatsiana Kupreichyk, Thomas Pauly, Matthias Stoldt, Wolfgang Hoyer, Antje Willuweit, Janine Kutzsche, Nils-Alexander Lakomek, Luitgard Nagel-Steger, Lothar Gremer, Gültekin Tamgüney, Jeannine Mohrlüder, Dieter Willbold

**Affiliations:** 1 Institut für Physikalische Biologie, Heinrich-Heine-Universität Düsseldorf, Düsseldorf, Germany; 2 Institute of Biological Information Processing (IBI-7), Forschungszentrum Jülich, Jülich, Germany; 3 Priavoid GmbH, Düsseldorf, Germany; 4 Institute of Neuroscience and Medicine (INM-4), Forschungszentrum Jülich, Jülich, Germany

**Keywords:** Drug development, Drug screening

## Abstract

A hallmark of Parkinson’s disease (PD) is the progressive neurodegeneration associated with soluble oligomeric and fibrillar forms of misfolded α-synuclein (α-syn). In this study, all-d-enantiomeric peptide ligands are presented that bind monomeric α-syn with high affinity, stabilize its physiological monomeric status, prevent aggregation and dissolve existing aggregates. This “antiprionic” mode of action directly targets pathogenic aggregated particles. Using mirror-image phage display on d-enantiomeric full-length α-syn, SVD-1 and SVD-1a were identified, showing a delay of aggregation and reduction of aggregate formation in both de novo and seeded models. Picomolar KDs were confirmed by SPR, where a highly dynamic interaction mode was verified by PRE-NMR. SVD-1a also reduced the toxicity and intracellular seeding of α-syn fibrils in cell culture by disassembling them into monomers, as confirmed by atomic force microscopy and dynamic light scattering. These results support SVD-1a as a promising lead compound for the treatment of Parkinson’s disease.

## Introduction

Fibrils consisting of α-syn have been extracted from the brain tissue of patients suffering from Parkinson’s disease (PD). PD, dementia with Lewy bodies (DLB) and multiple system atrophy (MSA) are diseases that are collectively referred to as synucleinopathies, as they are all characterized by the accumulation of insoluble α-synuclein (α-syn) aggregates in neuronal cells. The majority of the filamentous proportion of these deposits is composed of the α-syn protein^1,2^. α-Syn, encoded by the *SNCA* gene, is a 140 amino acid, 14.6 kDa, pre-synaptically located, and intrinsically disordered protein (IDP), which is thought to be involved in synaptic vesicle trafficking, synaptic plasticity, as well as modulation of neurotransmitter release, including dopamine^3,4^. More than insoluble α-syn fibrils, smaller soluble versions of them, as well as α-syn oligomers, are suspected to be responsible for the progression of the disease and the spreading of the pathology through the brain.

On- as well as off-pathway oligomers show a clear negative impact on many cellular processes, including membrane, proteasome, mitochondria and ER function, as well as inflammation, autophagy, and synaptic transmission^5–7^. In addition, recent insights suggest that aggregated species of α-syn are able to self-propagate between neuronal cells in a prion-like manner and, therefore, might be the central factor of the progressive nature of disease pathology^8–11^.

**Fig. 1:**
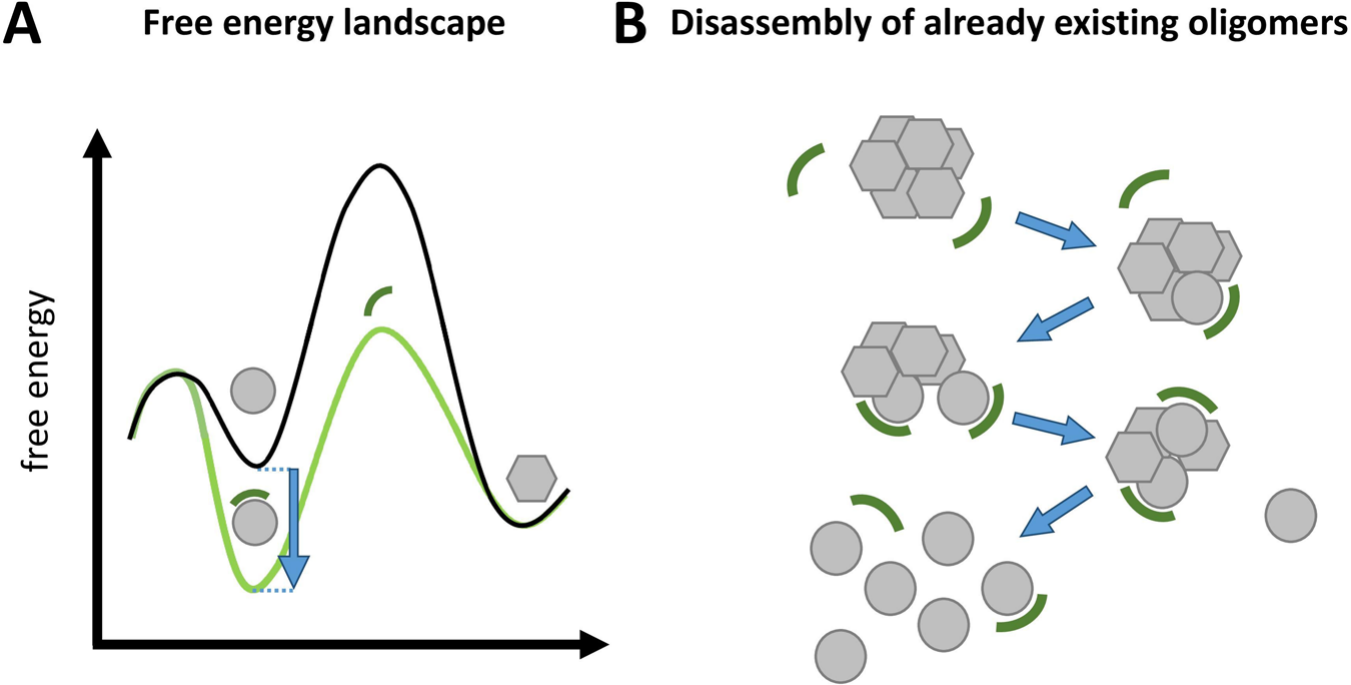
Mechanistic model of the anti-prionic mode of action realized by the all-d peptide described in the present work. Anti-prionic all-d-peptides are designed to stabilize monomers in their intrinsically disordered conformation—symbolized by circles. This conformation is distinct from the yet unknown, but certainly highly defined beta-sheet-rich conformation building blocks in oligomers—symbolized by hexagons. **A** Qualitative and schematic free energy landscape for the anti-prionic mode of action. The black line represents the energy landscape in the absence of the anti-prionic all-d-peptide. Monomer building blocks in oligomers are more stable than monomers. This allows the formation of oligomers from monomers thermodynamically, although there is a kinetic barrier, which is called primary nucleation and is currently under intensive investigation. Stabilization of the monomer by the anti-prionic all-d-peptide is lowering the free energy of the monomer (light green line) when in complex with the all-d-peptide, by the free binding energy (blue arrow) of the complex. Because in the presence of the anti-prionic all-d-peptide, the monomer has a lower free energy as compared to the oligomer, oligomers are disassembled into monomers. **B** Mechanistic model for disassembly of already existing oligomers from top to bottom: Anti-prionic all-d-peptides—symbolized by circle segments—approach oligomers. Due to their affinity to α-syn monomers, each all-d-peptide will interact with one of the α-syn building blocks within the oligomer assembly and thereby pushes its conformation towards the intrinsically disordered monomer conformation. This is incompatible with the oligomer assembly and therefore destabilizing the oligomer assembly. Further destabilization by interaction of additional anti-prionic molecules with other monomer building blocks ultimately leads to the complete disassembly of the oligomer into monomers in their intrinsically disordered conformation. Both molecules remain disordered in this transient complex, which may therefore be called “fuzzy complex”^31^. We called this mode of action “anti-prionic”, because it is ultimately disrupting prion-like behaving aggregates^12^.

In this sense, the most promising mode of action for PD treatment is the destabilization and direct elimination of the toxic and self-replicating α-syn species. We have therefore termed such a therapeutic strategy and the compounds that realize it, “anti-prionic”^12^. This term stays in analogy to the term “antibiotic” where elimination of the self-replicating pathological species is also the desired mode of action. In contrast to antibiotics, where it is about killing bacteria by chemical intervention of bacterial enzymes, anti-prionics need to reverse a thermodynamic equilibrium that favors the formation of α-syn fibrils and oligomers from α-syn monomers (Fig. 1). This is achieved by compounds that stabilize the IDP conformation of the α-syn monomer.

The success of this therapeutic strategy against the target amyloid-beta (Aβ) oligomers for the treatment of Alzheimer’s disease (AD) was previously demonstrated with the anti-prionic compound RD2^13–15^. RD2 is a 12-aa all-d-enantiomeric peptide that binds Aβ monomers with high affinity^16^. This results in the destabilization and ultimately the disassembly of oligomers into monomers. This kind of target engagement has been demonstrated in vitro^14^, in vivo^13^ and most recently, ex vivo with patient brain-derived Aβ oligomers^17^. Moreover, the compound proved cognitive restoration when administered to AD-mice with full-blown pathology^13^, while its d-enantiomeric structure and low molecular weight confer proteolytic stability, high bioavailability and penetration of the blood–brain barrier^14,18,19^.

In this study, we demonstrate the realization of the anti-prionic mode of action for α-syn. The developed all-d-enantiomeric peptides SVD-1 and SVD-1a bind monomeric α-syn with high affinity, inhibit non-seeded as well as seeded α-syn aggregation, and most importantly disassemble pre-formed fibrils (PFF) into monomers with high efficiency.

## Results

### Selection and optimization of α-synuclein-binding all-d peptides

**Fig. 2:**
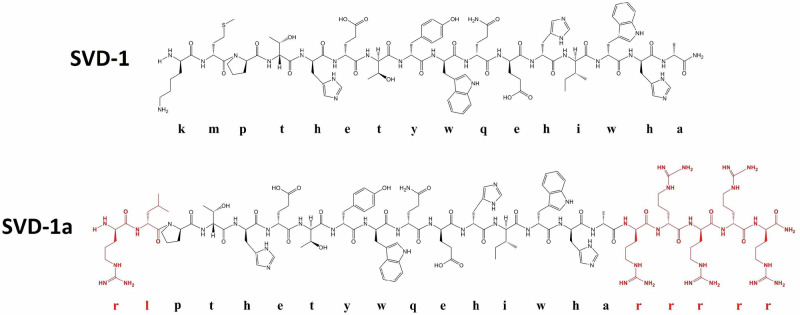
Natta projection of the d-enantiomeric lead compound SVD-1 and its first optimized derivative SVD-1a. Amino acid residues marked in red were exchanged or added for improved bioavailability, membrane penetrance and inhibitory effects.

A phage display selection against the mirror image of full-length α-syn yielded SVD-1 (Fig. 2) as the most promising all-d-peptide for stabilization of α-syn monomers (for full description of the phage display and peptide screening procedure, please see Supplementary Figs. 1–6).

SVD-1a is a derivative of SVD-1 in which d-methionine has been replaced by d-leucine at position 2 and d-lysine by d-arginine at position 1. These modifications were introduced to prevent possible alterations of these residues under physiological conditions, such as oxidation, acetylation, or phosphorylation, respectively. Five d-arginines were added at the C-terminal end to increase the overall solubility and potential membrane permeability^20^. Taken together, these modifications result in the sequence shown in Fig. 2.

### High-affinity binding of SVD-1 and SVD-1a to α-synuclein monomers

**Fig. 3:**
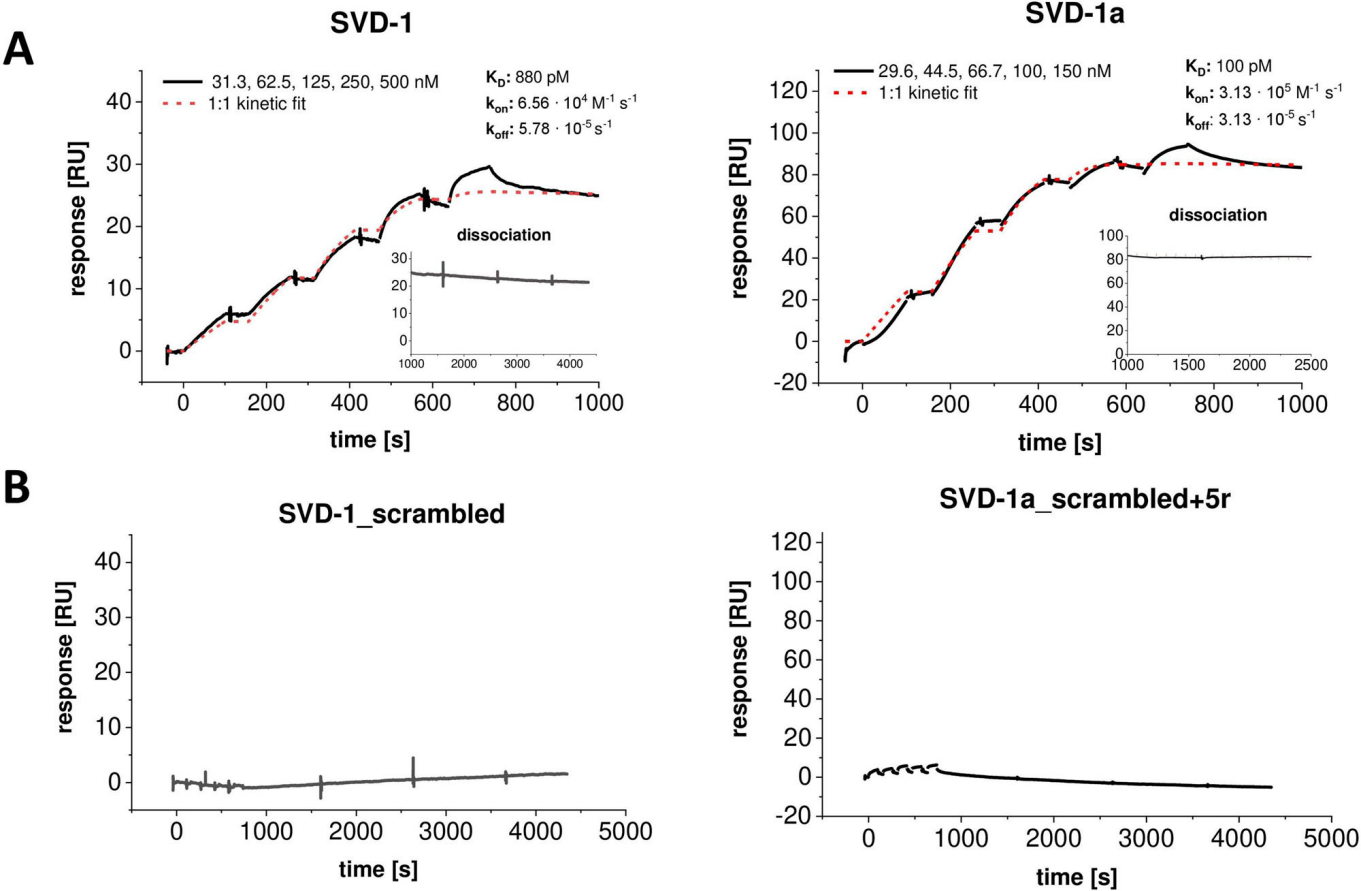
Single-cycle kinetic experiment with α-syn and immobilized SVD-1 and SVD-1a and control peptides. **A** SVD-1 (left) and SVD-1a (right) were immobilized on a carboxyl dextran matrix via amino coupling until saturation was reached (CMD200M, Xantec, GE). α-Syn was injected for 100 s at 30 µl/min in PBS 7.4 in a serial dilution ranging from 30 to 500 nM, followed by a dissociation time of 60 or 30 min, respectively. The experiment was performed as an individual measurement. The interaction kinetics were fitted with a 1:1 kinetic interaction model: SVD-1: *K*_D_: 880 pM, *k*_on_: 6.56 × 10^4 ^M^−1^ s^−1^, *k*_off:_ 5.78 s^−1^ × 10^−5^; SVD-1a: *K*_D_: 100 pM, *k*_on_: 3.13 × 10^5^ M^−1^ s^−1^, *k*_off_: 3.13 × 10^−5 ^s^−1^. The non-referenced signal of the active and referenced surface is shown in Supplementary Fig. 7. **B** SVD-1_scrambled (left) and SVD-1_scrambled+5r (right) were immobilized on a carboxyldextran matrix via amino coupling until saturation was reached (CMD200M, Xantec, GE). Full-length α-syn was injected for 100 s at 30 µl/min in PBS 7.4 in a serial dilution ranging from 15 to 250 nM, followed by a dissociation time of 60 min. *Y*-axis scaling was adjusted to (**A**).

First, we characterized the binding affinities of SVD-1 and SVD-1a to α-syn monomer. Surface plasmon resonance (SPR) experiments allow the detection of kinetic values, thereby giving insights into the time dependency of target recognition and complex rigidity. Prior to measurements, the SVD peptides were immobilized on a carboxyl dextran matrix surface via amino coupling, and α-syn was injected as analyte in the concentration range of 30–500 nM (Fig. 3A). Likewise, a control peptide that had the same amino acid composition as SVD-1 but with a random amino acid sequence (SVD-1_scrambled: ihyawtphtkhmqewe-NH2) was immobilized. As control for SVD-1a, five arginines were added to the C-terminus of the SVD-1 control peptide (SVD-1_scrambled+5r: ihyawtphtrhlqewerrrrr-NH2; (Fig. 3B)).

When applying a 1:1 Langmuir-based interaction model, we obtained a *K*_D_ value of 880 pM with a *k*_on_ of 6.56 × 10^4^ M^−1^ s^−1^ and a *k*_off_ of 5.78 × 10^−5^ s^−1^ for SVD-1. For SVD-1a, a *K*_D_ of 100 pM with a *k*_on_ 3.13 × 10^5^ M^−1^ s^−1^ and a *k*_off_ of 3.13 × 10^−5^ s^−1^ was identified. However, the data is not fully explained by the 1:1 model, which is evident from a deviation of the fits from the data obtained for the later injections, suggesting an additional low-affinity binding mode in the higher nM range.

### Inhibition of α-synuclein seeded and de novo aggregation

The inhibitory effects on amyloid formation were further verified in ThT assays under de novo as well as seeded conditions with several ratios of α-syn and SVD-1 or SVD-1a (Supplementary Figs. 8 and 9). For seeding assays, we incubated SVD-1 or SVD-1a together with small α-syn pre-formed fibrils (PFF) that induce fibril mass growth in the presence of monomeric α-syn. To obtain fibril seeds with a homogeneous size and high seeding capacity, we started from mature fibrils and treated them with harsh ultrasonication. Any remaining larger fibrils were removed by ultracentrifugation as described by Kaufmann et al. ^21^.This yielded short fibrils with a high ratio of fibril ends per mass as shown by AFM (Supplementary Fig. 10). Referring to their small size, we call them “small α-syn PFF seeds” throughout the manuscript. Due to their high ratio of fibril ends per mass, added monomeric α-syn is very efficiently and reproducibly elongating the seeds^22^.

The sequence specificity of the effect was confirmed by comparing the inhibitory effects of SVD-1 and SVD-1a with the respective control peptides. The sequence-randomized control peptides had virtually no inhibitory effects, clearly suggesting that neither the overall electrostatic charge nor hydrophilicity was most important, but the amino acid sequence of SVD-1 and SVD-1a (Supplementary Figs. 8 and 9). In contrast to the sequence-randomized control peptide, SVD-1 confirmed its efficacy by a concentration-dependent delay of aggregation onset as well as by a reduction of steady state levels under de novo aggregation conditions. Thus, SVD-1 inhibits primary nucleation of α-syn. Also, in contrast to the sequence-randomized control peptide, SVD-1 was able to significantly decelerate amyloid growth in the seeded environment. Thus, SVD-1 also inhibits elongation and secondary nucleation of α-syn. Similar observations were made for SVD-1a under de novo aggregation conditions, where at twofold molar excess aggregation was completely inhibited. When SVD-1a was present in the pre-incubation period with the small α-syn PFF seeds, a reduction of the elongation rate was observed later during the incubation period with α-syn monomers already at 5 µM SVD-1a. 20 µM or higher SVD-1a concentrations even led to the complete inhibition of the seeding capacity. 20 µM of the control peptide, SVD-1_scrambled+5r, did not yield any inhibition of seeding, again underlining the SVD-1a sequence specificity for the observed effects.

### Disassembly of small α-synuclein pre-formed fibril (PFF) seeds by SVD-1a

**Fig. 4:**
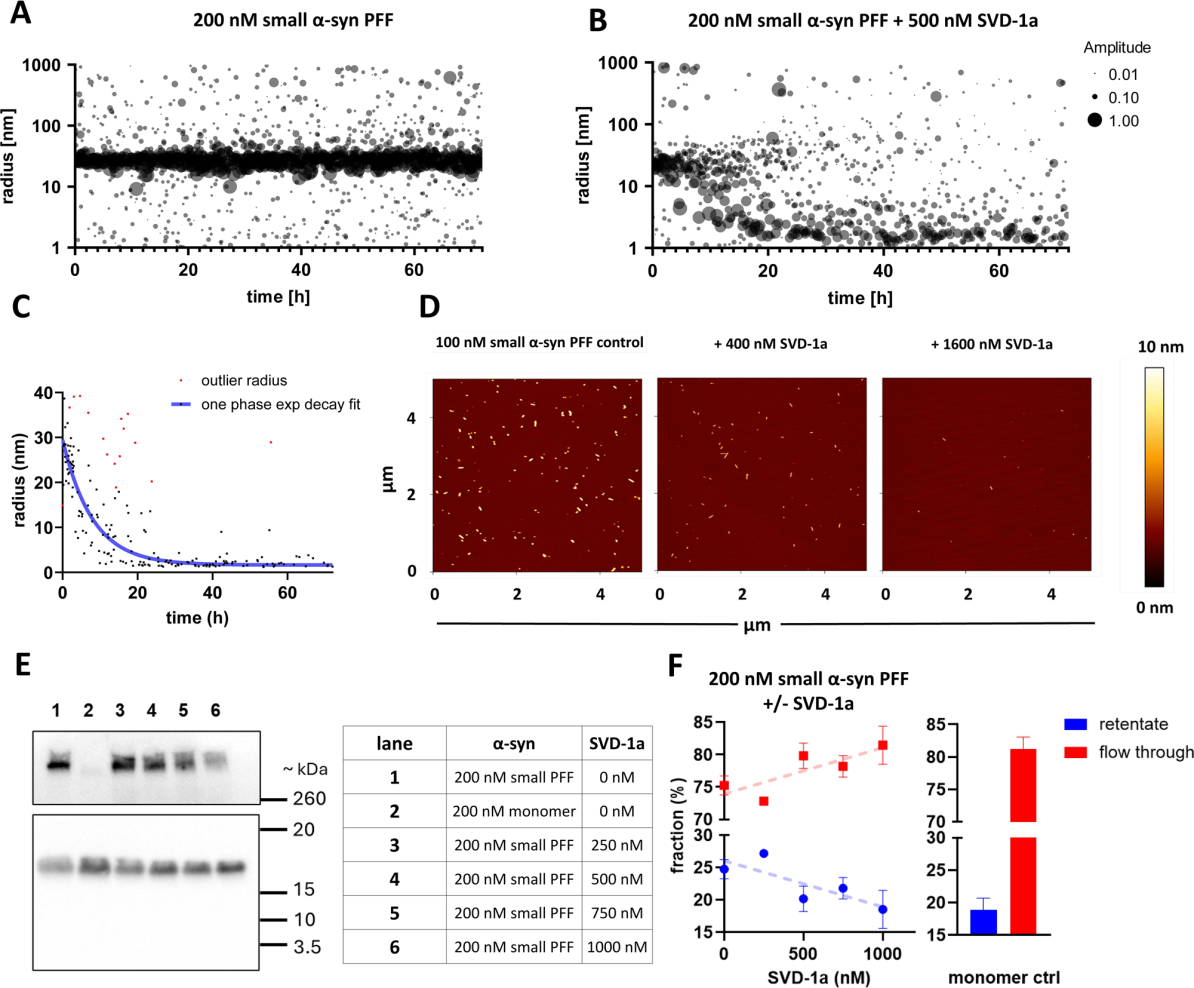
SVD-1a disassembles small α-syn PFF seeds into α-syn monomers. Small α-syn PFF seeds were prepared as described previously. 100 or 200 nM monomer equivalent small α-syn PFF seeds were incubated with or without 400, 500, 1600 nM SVD-1a for 3 days at 37 °C in PBS pH 7.4. **A** and **B** Time-dependent DLS measurements with 200 nM small α-syn PFF seeds in the absence (**A**) and presence (**B**) of 500 nM SVD-1a. 1 ml sample was continuously measured in a sealed quartz cuvette at 37 °C under quiescent conditions every 60 s for 72 h in a SpectroSize 300 instrument (XtalConcepts, GE). Data are shown as a radius plot where the signal amplitude of each particle size is represented by the data point diameter. **C** One-phase exponential decay fit of “200 nM PFF + 500 nM SVD-1a” shown in (**B**). Fit was performed for particles in the range of 1–100 nm with an amplitude > 0.2. Outliers were excluded from fitting using a *Q* value of 1%. Fitting results: Span = 23.38 nm, rate constant = 0.132 1/s; half-life = 5.2 h. Fitting was performed using the “One phase exponential decay” fitting function from GraphPad Prism 10 (GraphPad Software Inc., USA). SVD-1a does not precipitate small α-syn PFF seeds as demonstrated by protein quantification after centrifugation, shown in Supplementary Fig. 12. **D** For AFM analysis, small α-syn PFF seeds pretreated with or without 400 and 1600 nM SVD-1a were incubated and dried on a freshly cleaved mica surface, followed by washing with ddH_2_O and drying using a gentle stream of N_2_. Analysis was performed using the NanoWizard 3 system (J-1100, JPK BioAFM, USA), recording multiple surface sections. The sections shown are representative of the observed species and particle density identified on all surface sections. **E** Exemplary Western blot of small α-syn PFF samples incubated with SVD-1a. 200 nM small α-syn PFFs (monomer equivalent) were incubated with increasing concentrations of SVD-1a for 72 h at 37 °C. PFF and monomer samples that were incubated without SVD-1a served as controls. α-Syn was detected with the antibody Syn211. A prominent α-syn monomer band was present at the expected molecular weight. Below the monomer bands, no further bands were visible. In the upper part of the blot, above the marker range, PFFs were detected, using a longer exposure time due to a weaker signal (see Supplementary Fig. 11 for the whole blot image with different exposure times). **F** Size-based fractionation by centrifugal concentration followed by ELISA quantification. 200 nM small α-syn PFF (monomer equivalent) were incubated with or without SVD-1a together with a 200 nM monomer control for 72 h at 37 °C. After incubation, the samples were fractioned using a 100 kDa MWCO centrifugal concentrator (Merck, Microcon DNA Fast Flow 100 MWCO). α-Syn content of flow through (red) and retentate (blue) was quantified using an α-syn specific ELISA (BioLegend, Human α-Synuclein (Colorimetric), 448607). Shown are the fractions of the retentate and flow through as a percentage of the total concentration identified for each sample. A linear regression line (blue or red dotted lines) was included for the SVD-1a-treated samples (left) to underline the concentration dependency of the SVD-1a treatment effect. The fractions identified for the α-syn monomer control sample are shown as a bar graph (right). Notably, the sample treated with 1000 nM SVD-1a shows the same distribution as the monomer control. Data are shown as mean values with ±SD (*n* = 3).

It is tempting to speculate that SVD-1a was successfully reducing the amount of small α-syn PFF seeds during the 20 h pre-incubation period to explain the reduction of their seeding activity. To investigate this further, we incubated 100 or 200 nM monomer equivalent purified small α-syn PFF seeds with increasing concentrations of SVD-1a for 72 h and analyzed the samples by time-dependent dynamic light scattering (DLS), atomic force microscopy (AFM), Western blot and *M*_W_-based membrane fractionation (Fig. 4).

Time-dependent DLS measurement (Fig. 4A and B) shows that SVD-1a progressively eliminates the small α-syn PFF seeds (Fig. 4B, ~26 nm radius) by disassembling them into monomeric α-syn (Fig. 4B, ~1.6 nm radius). In contrast, no change in the small α-syn PFF particle size radius was observed when SVD-1a is absent (Fig. 4A, 26 nm radius). A mono-exponential fit was identified as the best fit for describing the degradation of small α-syn PFF seeds to monomers by SVD-1a (Fig. 4C). Incubation of the small α-syn PFF seeds with increasing concentrations of SVD-1a (400 and 1600 nM) also resulted in a decrease in small α-syn PFF particle number as supported by AFM (Fig. 4D). Destabilization of small α-syn PFF and conversion into monomers is confirmed by a Western blot that specifically detects α-syn (Fig. 4E). The intensity of higher *M*_W_ bands, which are exclusively present in small α-syn PFF samples (comparison to monomer control), is progressively reduced in presence of higher SVD-1a concentrations (see Supplementary Fig. 11 for whole blot image). Accordingly, *M*_W_-based fractionation with 100 kDa MWCO^23^ followed by ELISA quantification (Fig. 4F) shows an SVD-1a concentration-dependent decrease of the α-syn fraction in the retentate—corresponding to small α-syn PFF—and an increase in the eluate, indicating a destabilization of α-syn small PFF by stabilization of the α-syn monomers. It is important to note that incubation of small α-syn PFF seeds with SVD-1a did not lead to precipitation of these species, but to stabilization of the monomers. This was demonstrated by measuring the protein concentration in the supernatant and in the pelleted fraction after centrifugation at different incubation time points (Supplementary Fig. 12). Moreover, elimination of the small α-syn seeds by SVD-1a leads to stabilization of the random coil secondary structure of the pre-existing non-folded α-syn monomer, while in the absence of the compound, a conversion to beta-sheet absorption spectra occurs, which is a typical indicator of fibril formation (Supplementary Fig. 13).

In conclusion, SVD-1a stabilizes monomeric α-syn in its random coil status and demonstrates its anti-prionic mode of action by disassembling pre-existing small α-syn PFF seeds into α-syn monomers in a time and concentration-dependent manner.

### Inhibition of intracellular seeding and cytoprotection

**Fig. 5:**
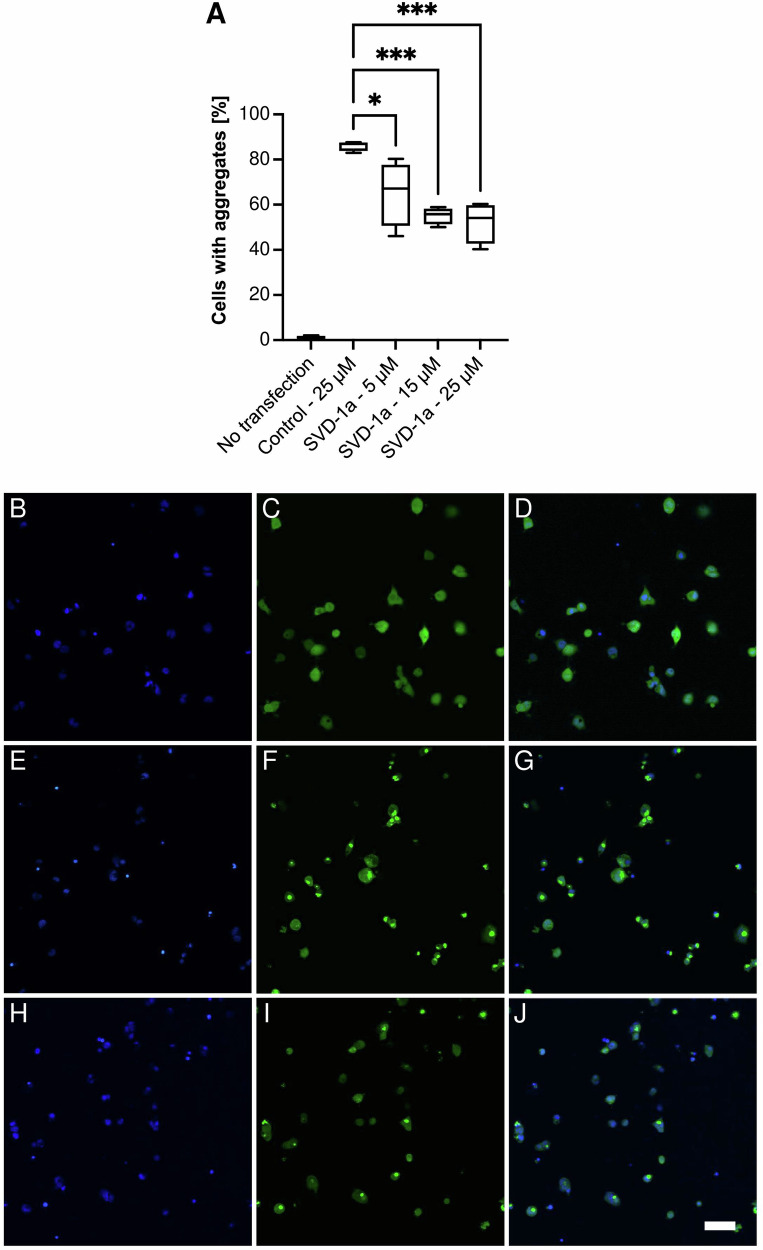
SVD-1a inhibits seeded α-syn aggregation in cells. To validate the inhibitory effect of SVD-1a in cells, we used the α-synA53T–YFP cell system, which stably expresses human α-syn with the familial A53T mutation fused to YFP in HEK293T cells and enables fluorescence-based detection of aggregates that show up as highly fluorescent spots within the cells, clearly above background, intracellularly after seeding with small α-syn PFF seeds. In order to avoid interference of the peptide with the uptake of small α-syn PFF seeds, we performed a two-step transfection starting with a first transfection of the peptide and a second transfection with PFFs. SVD-1a but not the negative control peptide (kgvgnleyqlwalegk-NH2) inhibited α-syn aggregation in αSynA53T–YFP cells in a dose-dependent manner (**A**). To quantify the number of cells with aggregates in our images and to avoid experimental bias, we used a fully automated algorithm for image analysis. Significance was calculated using one-way ANOVA followed by Dunnett’s multiple comparisons test. One asterisk denotes *p* values < 0.05 and three asterisks a *p* value < 0.001. Error bars indicate standard deviation. In contrast to unseeded cells, which did not harbor any α-syn aggregates at day 3 after plating (**B-D**), seeding with soluble α-syn PFFs-induced aggregation in 85% of cells treated with a negative-control peptide (25 µM) that does not bind to α-syn (**E-G**). Treatment with increasing concentrations of SVD-1a, here imaged at 25 µM, led to a concentration-dependent reduction in the number of cells with aggregates (**H-J**). Panels **B**, **E**, and **H** show nuclei stained with Hoechst 33342. Panels **C**, **F**, and **I** show αSynA53T–YFP fluorescence. Panels **D**, **G**, and **J** show merged images. The scale bar in J represents 100 µm and applies to all panels. The viability of the cells was not reduced by transfection with seeds or in SVD-1a, as shown in Supplementary Fig. 15. Confirmation that highly fluorescent aggregates contain α-syn is shown in Supplementary Fig. 14 by co-immunostaining with syn-211 antibody, anti-α-syn (phospho S129) antibody and 5G4 antibody.

To further verify whether SVD-1a is also able to counteract the seeding potential of soluble α-syn small α-syn PFF seeds in living cells, α-synA53T–YFP-expressing cells were transfected with soluble α-syn small α-syn PFF seeds together with SVD-1a or a negative-control peptide with similar molecular weight as SVD-1a but no affinity to monomeric α-syn (Fig. 5). In the α-synA53T-YFP cell system, human α-syn with the familial A53T mutation fused to YFP is stably expressed in HEK293T cells and enables fluorescence-based detection of intracellular aggregation of endogenously expressed α-synA53T after seeding with patient-brain extracts containing PFFs as shown previously^24–27^ or with small α-syn PFF seeds as shown here. To avoid interference with the fluorescence-based aggregate detection, we intentionally did not use fluorescence-labeled SVD-1a or the control peptide. Another reason was that fluorescent dyes can positively or negatively interfere with the transfection efficacy and possibly also with the subcellular localization of the respective peptide.

Seeding with small α-syn PFF seeds in the presence of a negative-control peptide induced aggregation in 85% of the cells (Fig. 5A, E–G). We verified that the induced intracellular, yellow-fluorescent aggregates consist of α-syn by immunofluorescence staining with three different antibodies recognizing aggregated α-syn, α-syn phosphorylated at serine 129, and total α-syn (Supplementary Fig. 14). When SVD-1a was transfected into the cells, a concentration-dependent reduction of aggregate-positive cells was observed, resulting in 64% and 57% aggregate-positive cells using 5 and 25 µM of SVD-1a, respectively (Fig. 5A, H–J). Also, the viability of PFF-seeded α-synA53T–YFP cells was not significantly reduced in the presence of any of the two peptides (Supplementary Fig. 15). SVD-1aCys_Alexa647 crosses the membrane without transfection (Supplementary Fig. 16). These results demonstrate that SVD-1a inhibits PFF-induced aggregation of α-syn in the intracellular environment without inducing cytotoxicity. Moreover, in PC12 cells a reduction of PFF cytotoxicity was verified in the presence of SVD peptides using cell viability assay (Supplementary Fig. 17).

### SVD-1a interacts in a highly dynamic interaction mode with disordered α-synuclein monomers

SVD-1 and SVD-1a have been selected and developed to specifically bind α-syn monomers in order to stabilize α-syn in its monomeric conformation. The constant and fast sampling of a large conformational space gives IDPs the structural plasticity and adaptability to interact with and control multiple binding partners at the same time. IDPs have very characteristic NMR spectra. The amide protons of the protein backbone are solvent-exposed and not involved in typical secondary structural elements like β-sheets or α-helices. This and the high mobility of the protein backbone and the side chains on a very rapid time scale limit the chemical shift dispersion of IDPs more or less to the time-averaged random coil chemical shifts of the respective amino acid residues of the protein in aqueous solution. This is the reason why the amide protons of IDPs have a typical chemical shift dispersion of only 0.7 ppm. In contrast, the amide protons of globularly folded proteins have a chemical shift dispersion of up to 4 ppm, and the individual chemical shift of the amide proton is dependent on its involvement in a hydrogen bond and whether its chemical environment contributes more shielding or de-shielding contributions, both of which are not averaged out over time as in IDPs^28,29^.

**Fig. 6:**
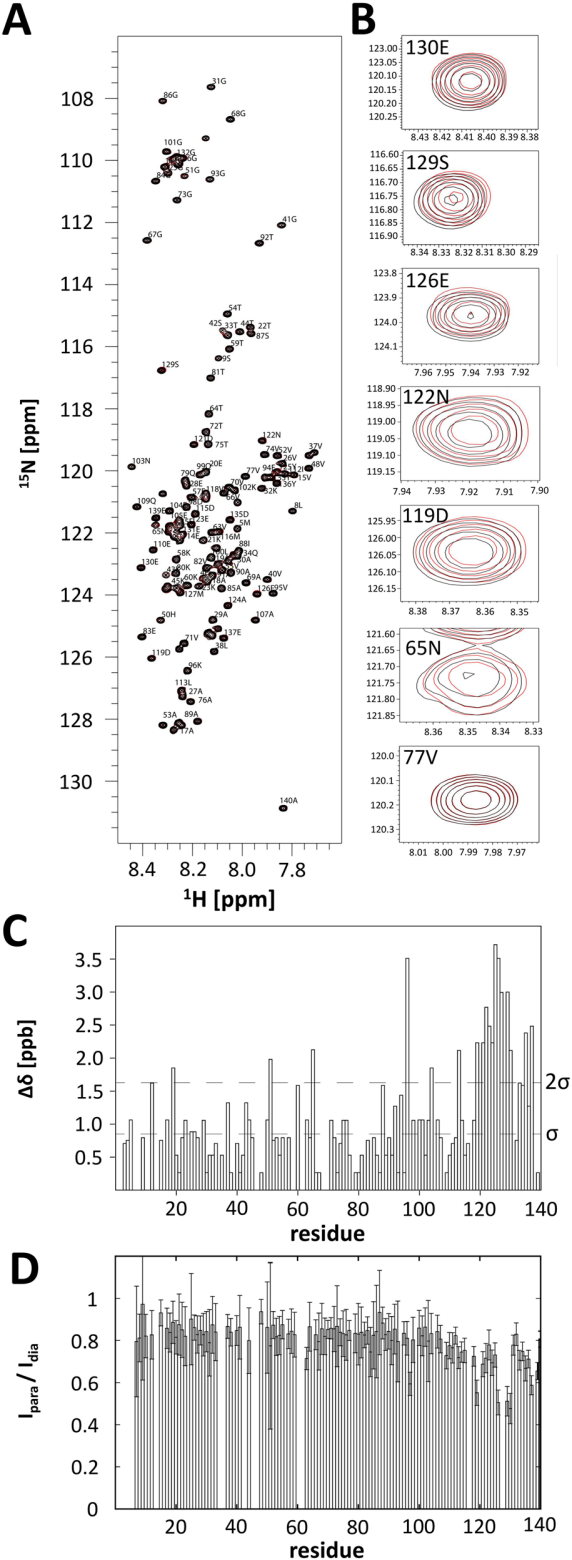
NMR analysis of ^15^N-labeled α-syn interacting with SVD-1a (not isotope labeled). **A** Overlay of two-dimensional ^1^H-^15^N HSQC spectra of 25 µM ^15^N α-syn in the presence (red) and absence (black) of an equimolar amount of SVD-1a. **B** Enlargement of several resonances in the spectra shown under (A) that show small chemical changes for residues in the presence (red) relative to the absence (black) of SVD-1a (130E, 129S 126E, 122N, 119D, 65N); for comparison, resonance 77 V is shown that does not show any chemical shift change. **C** Residue-specific absolute NMR chemical shift changes in the spectra of ^15^N α-syn in the presence of SVD-1a relative to the absence of SVD-1a. The standard deviation, σ, as well as the 2-fold standard deviation, 2*σ*, of the distribution of observed chemical shift changes are indicated as dashed lines. **D** NMR PRE intensity ratios of ^15^N α-syn in the presence of the paramagnetically-labeled SVD-1a. Residue-specific intensity ratios, *I*_para_/*I*_dia_, of the cross-peak intensities in the two-dimensional ^1^H–^15^N NMR spectra of the paramagnetic vs. diamagnetic sample are shown. The lower the intensity ratios, *I*_para_/*I*_dia_, the closer the proximity of the paramagnetically-labeled SVD-1a to the respective residue of α-syn. An intensity of one would indicate the absence of interactions. Data point to a bit more pronounced (transient) binding interaction of SVD-1a with residues in the C-terminal region of α-syn, as compared to the average effect on residues in the remaining N-terminal region.

Next, we wanted to investigate whether SVD-1 and SVD-1a have a significant impact on the IDP conformation of α-syn monomers, given their high affinity based on SPR. The highly dynamic and transient interactions may, for each amide proton and ^15^N-nucleus, lead to many different shielding and de-shielding events in their chemical environment that become zero when averaged over the NMR time scale. The probability, however, that each chemical shift change is averaged to exactly zero, is very low. Thus, we investigated the chemical shift changes upon binding of SVD-1a to α-syn monomers at the highest available field. Figure 6A shows the superposition of the ^1^H-^15^N HSQC NMR spectra of 25 µM ^15^N-labeled full-length α-syn in the absence and presence of 25 µM SVD-1a. Careful and automated peak analysis revealed that there are indeed small chemical shift changes that are shown in the α-syn sequence, specifically in Fig. 6C, with some examples displayed in Fig. 6B. Overall, the chemical shift changes are very small, with most residues showing significant chemical shift changes located in the C-terminal region, but also residues in other parts of α-syn are affected.

To obtain more information about which parts of the α-syn molecule are involved in the highly dynamic and transient interaction, we applied paramagnetic relaxation enhancement (PRE) NMR experiments. Such NMR-PRE data have proven insightful for the study of binding interactions of amyloid-β recently^30^.

Residue-specific NMR PREs intensity ratios, *I*_para_/*I*_dia_, were recorded for ^15^N-labeled (NMR-visible) α-syn in the presence of SVD-1a, with a paramagnetic spin-label covalently attached at its C-terminus (Fig. 6D). Decrease in intensity ratios points to the proximity of the paramagnetic spin-label of SVD-1a to the respective residue of α-syn. Indeed, we observed an overall decrease of intensity ratios, *I*_para_/*I*_dia_, in the spectra (paramagnetic vs. diamagnetic sample) for practically all residues, in the order of 15–20% and with an increased reduction for the C-terminal residues of α-syn (Fig. 6D). Strikingly, this coincides with the residues showing the “largest” of the very small chemical shift changes (Fig. 6B) that were most prominent in the C-terminal region. In order to investigate whether SVD-1a has any sequence similarity with parts of α-syn, we carried out a sequence comparison (Supplementary Fig. 18). Although there is a small similarity of SVD-1a with a C-terminal sequence stretch, in our view, the sequence similarity is not high enough to convince us of its relevance for the mechanism of interaction. Another argument is that although the interaction takes place with a slight C-terminal emphasis, it was shown by chemical shift perturbation and PRE-NMR (Fig. 6) that SVD-1a is binding along the entire primary sequence of α-syn. The apparently weak transient interactions with the N-terminal 108 α-syn residues, as observed by the NMR-PRE data, are in their accumulated sum effect relevant and impactful. This is indicated by the observation that the de novo aggregation not only of full-length α-syn is efficiently inhibited by SVD-1 and SVD-1a, but also that of the C-terminal deletion mutant of α-syn, α-syn (1–108) (Supplementary Fig. 19).

The PRE data, together with the absence of large chemical shift changes, are in line with lowly populated transient binding interactions, presumably due to an on/off hopping of SVD-1a to α-syn occurring on a very fast time scale. Hence, the binding mode is potentially best described by an IDP–IDP interaction with multiple dynamic binding sites, whereas both partners retain their disordered structure^31,32^. Such fuzzy complexes have been described previously^33–39^. In some cases, no chemical shift changes have been reported at all^33^. For several of those cases, NMR paramagnetic relaxation enhancement (PRE) measurements revealed transient interactions between the protein and the binding partner^33–39^.

### SVD-1a stabilizes α-Syn monomers under strong aggregation-promoting conditions

To investigate in further detail how this interaction mode functions under aggregation-promoting conditions, we performed de novo aggregation in the presence and absence of the peptide and analyzed the endpoint samples by circular dichroism spectroscopy (CD) and AFM. In addition, at the end of the incubation period, samples were separated into monomeric and aggregated fractions using centrifugal membrane filters with a 100 kDa molecular weight cut-off. These fractions were subsequently analyzed by SDS–PAGE and α-syn-specific ELISA.

**Fig. 7:**
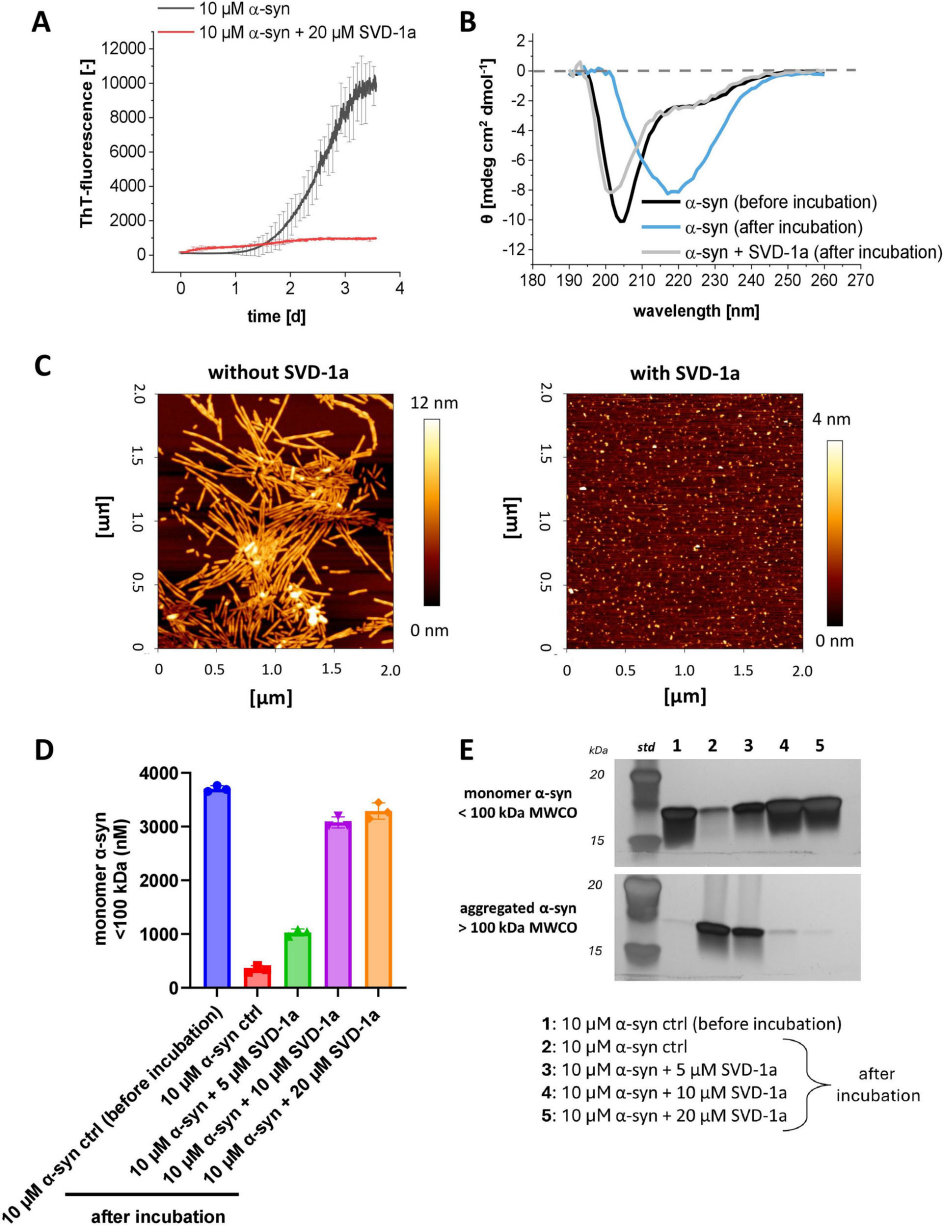
De novo aggregation analysis of α-syn in the presence and absence of SVD-1a. **A** De novo ThT assay of 10 µM α-syn with and without 20 µM SVD-1a. ThT fluorescence progression was measured in a 96-well non-binding half-area plate (Corning, USA) with a FLUOStar plate reader (BMG labtech, GE) at *λ*_ex_ = 448 nm and *λ*_em_ = 482 nm with 300 rpm continuous orbital shaking between reads. For induction of aggregation, one borosilicate bead per well was added (*d* = 3 mm, Hilgenberg, GE). Data are shown as mean values with ±SD (*n* = 5). **B** CD secondary structure analysis of de novo aggregation samples. Samples were incubated as described in (**A**) without added ThT (*n* = 3) and subsequently pooled for CD analysis. Far-UV ellipticity of the samples was measured in a quartz cuvette (*l* = 10 mm) in a J-1100 CD-spectrometer (Jasco, GE). In addition to (**A**), a sample with 20 µM SVD-1a alone was incubated under identical conditions and later used as a reference for the sample with α-syn and SVD-1a. For this sample (α-syn + SVD-1a (after incubation)), the SVD-1a reference subtracted CD spectrum is shown. **C** Samples from (**A**) were isolated directly after incubation and diluted in PBS pH 7.4 to a final concentration of 1 µM α-syn monomer equivalent. 5 µl diluted sample was incubated and dried on a freshly cleaved mica surface, followed by washing with ddH_2_O and drying using a gentle stream of N_2_. Analysis was performed using the NanoWizard 3 system (J-1100, JPK BioAFM, USA), recording multiple surface sections. The sections shown in (**C**) are representative of the observed species identified on all surface sections. **D** and **E** 10 µM α-syn monomer was incubated—analogous to the samples in (**A**)—with or without 5, 10, or 20 µM SVD-1a for 7 days (*n* = 7). The replicates were then united and loaded onto a 100 kDa MWCO centrifugal concentrator. In addition, a 10 µM α-syn sample was prepared and subjected directly to size-based fractionation. **D** α-Syn in the flow through (MWCO < 100 kDa) was quantified using an α-syn specific ELISA (BioLegend, Human α-Synuclein (Colorimetric), 448607, *n* = 3). **E** Retentate and flow-through samples were analyzed using SDS–PAGE with silver staining. The whole gel is shown in Supplementary Fig. 20.

CD secondary structure analysis (Fig. 7B) of replicate samples without ThT shows that incubation of monomeric α-syn results in a shift from random coil (Fig. 7B, black line) to beta-sheet (Fig. 7B, blue line) CD spectrum, typical for α-syn fibrils^40,41^. However, in the presence of SVD-1a, while the overall signal is slightly reduced, no spectrum shift towards a beta-sheet spectrum was observed (Fig. 7B, gray line). In combination with the ThT measurements, the CD measurements clearly indicate that the majority of monomeric α-syn that is present at the start point of incubation is retained in its monomeric random coil form when SVD-1a is present and is only aggregating into β-sheet-rich fibrils when SVD-1a is not present. These observations are confirmed by AFM pictures of the samples taken from the incubation endpoints in the ThT assay (Fig. 7C). Here, the sample without SVD-1a shows mature fibrils (Fig. 7C, left). The AFM images of samples containing SVD-1a reveal small particles that are likely artifacts resulting from the drying process (Fig. 7C, right). However, they clearly do not contain any fibrils, supporting that SVD-1a was able to inhibit fibril formation as already demonstrated by the ThT experiments. At the end of the incubation, samples were fractionated using 100 kDa MWCO centrifugal concentrators in the presence of increasing concentrations of SVD-1a. The eluate, representing the monomeric α-syn fraction, was quantified via α-syn-specific ELISA (Fig. 7D). In the pre-incubated control, ~3.7 µM α-syn was detected in the flow-through. In contrast, the incubated sample without compound showed a markedly reduced monomer concentration (~0.3 µM), as most α-syn had aggregated into species >100 kDa, retained by the filter. Addition of SVD-1a (5, 10, 20 µM) at the beginning of the incubation resulted in concentration-dependent stabilization of monomeric α-syn, with 20 µM restoring monomer levels in the eluate to those of the non-pre-incubated control (before incubation). SDS–PAGE analysis corroborated these findings (Fig. 7E). Without SVD-1a, the majority of α-syn remained in the retentate (Fig. 7E, lane 2). Notably, high-molecular-weight aggregates (>100 kDa) were not fully monomerized by SDS and heat treatment and accumulated near the gel pocket (Supplementary Fig. 20). With increasing SVD-1a concentrations, more α-syn appeared in the eluate (Fig. 7E, lanes 3–5), while aggregate levels in the retentate decreased. At 20 µM SVD-1a, the aggregate band intensity was substantially diminished, indicating effective stabilization of monomeric α-syn in the presence of the compound. These results support the observation of other experiments that SVD-1a stabilizes α-synuclein in its random coil conformation and inhibits the formation of β-sheet-rich fibrils.

## Discussion

In order to integrate the results shown into a mechanistic model (Fig. 1) of a therapeutic mode of action, the following considerations are crucial: only very small chemical shift changes were identified for unbound vs. bound α-syn, indicating that the presence of the compounds did not significantly change the overall α-syn conformation. At first glance, this may seem counterintuitive when compared to the classical receptor–ligand interaction studies, where one would expect chemical shift changes for the residues close to the “binding pocket”. However, the binding mode of IDPs, such as α-syn, may substantially deviate from such a classical picture^42,43^. Thus, we carried out NMR experiments with ^15^N isotope-labeled α-syn and SVD-1a with a paramagnetic label attached. When applying paramagnetic relaxation enhancements (PRE) experiments, the PRE-label associated with the peptide decreases the peak intensities around the binding site. As our PRE measurements indicate (Fig. 6D), SVD-1a interacts with the entire α-syn monomer, with the C-terminal part showing the strongest interaction. PRE data indicate a transient and highly dynamic interaction.

The binding data would be consistent with the following scenario: SVD-1 and SVD-1a encounter α-syn in various conformations. Each conformation of this conformational ensemble is transient and sparsely populated, with conformations interconverting among each other on fast time scales. In sum, the accumulated high number of sparsely populated binding events will result in the observed macroscopic high-affinity binding constant. Note that the observed overall binding will not necessarily induce large chemical shift changes for the individual residues^33,34^, but leads to the observed NMR PRE effects. Such interactions have been described before, e.g., in the modification of histones, where this type of interaction enables rapid switching between different states^31^. Although this is also a fuzzy complex consisting of two IDP interaction partners, a pM K_D_ was determined for the entire interaction, similar to what we observe here for SVD-1a and α-syn monomer. Also, one has to keep in mind that the extent of chemical shift perturbation is not a measure of affinity but a measure of change in the chemical environment of the observed nucleus. The time-averaged chemical environments of the observed amide groups, however, are mostly solvent for an IDP, in the absence or presence of SVD-1a. This is a binding mode, which comes closest to the envisioned mode of action for SVD-1 and SVD-1a, namely to stabilize α-syn monomers in their highly dynamic and flexible IDP conformation and to protect them from accumulation into toxic and seeding potent α-syn aggregates. The SPR results comparing SVD-1a and SVD-1a_scr.+5r (Fig. 3), as well as the comparison of the same compounds in the seeded assay (Supplementary Fig. 8), also show that the binding and compound effects are sequence-specific. However, since the SVD-1a control peptide shows improved effects compared to the parental sequence SVD-1 in the seeded assay, it is possible that improved effects are partly due to the mere introduction of additional positive charge in the form of arginines.

For the majority of the α-syn anti-aggregation compounds that have been developed over the last years, an essential dogma was the avoidance of a direct interference with the physiological monomeric form of α-syn in order to exclude effects that might influence the physiological function of the target protein^44,45^. However, recent studies have shown that the endogenous catalytic elimination of α-syn aggregates occurs via the binding and stabilization of monomer units^46^. The following data support the conclusion that SVD-1a also binds to the α-syn monomer, stabilizes it in its IDP-like conformation, and keeps it monomeric in its physiological IDP conformation. Incubation of 25 µM α-syn with 25 µM SVD-1a for 4.8 h at 10 °C for the NMR experiments shown in Fig. 6A did not yield any signs of signal loss due to precipitation. Similarly, for the PRE NMR experiments (Fig. 6D), the observed amide cross-peak intensity reduction due to the PRE label interaction with α-syn residues was fully rescued upon addition of ascorbate to quench the PRE label (which was the diamagnetic reference experiment). Thus, SVD-1a is not sequestering α-syn monomers into any other conformation or state. SVD-1a is rather stabilizing α-syn monomers in their IDP conformation by the free binding energy underlying the high affinity demonstrated by the SPR measurements (Fig. 3). The strong binding of SVD-1a to α-syn monomers is not influencing its IDP conformation, suggesting that its physiological role in the cell might not be affected or limited by SVD-1a. This assumption is further supported by the de novo aggregation experiments presented in Fig. 7, in which SVD-1a, at a two-fold molar excess, fully stabilizes monomeric α-syn in its random coil secondary structure after 7 days of incubation under aggregation-promoting conditions. Figure 1A illustrates why the stabilization of α-syn monomers by the free binding energy of SVD-1a is also disassembling already existing small α-syn PFF seeds (Fig. 1B), just because SVD-1a-bound α-syn monomers are thermodynamically more stable than the α-syn building block conformation PFFs. This strongly supports the proposed mode of action of SVD-1a. Incubation of small α-syn PFF seeds with SVD-1a led to the decrease of small α-syn PFF particle size and number as verified by DLS, AFM, Western Blot and size-based centrifugal membrane fractionation (Fig. 4). At the same time, the formation of monomer was detected (Fig. 4B, C, E, F). All these results support the mechanistic model for SVD-1a’s mode of action as described in Fig. 1.

Taken together, the small α-syn PFF seed elimination assay as well as the assays for which small α-syn PFF seeds were used as the pre-formed aggregate species (cell viability assay, intracellular aggregation assay, seeded aggregation assay) show that the compounds are able to eliminate soluble α-syn aggregates independent of their overall structural assembly. This result appears to be in agreement with the intended anti-prionic mode of action, where the compounds stabilize the physiological IDP-like monomer structure, thereby destabilizing and disassembling the toxic aggregates. This allows the mode of action to be independent of the specific conformation of specific toxic aggregate assemblies. The physiological solution structure of monomeric α-syn remains the same in vitro and in vivo, irrespective of the localization^47^. This makes monomeric α-syn a more attractive target, since this mode of action is independent of the final form of the toxic component, and thus independent of any prion strain.

These results are a promising starting point for further development of an all-d-enantiomeric peptide compound that disassembles already existing aggregates. Since the compounds presented here are predominantly interacting with the physiological active monomeric form of α-syn, future studies will also address the preservation of its physiological functionality in presence of the compounds. In addition, future efforts will deal with the investigation of the compound’s blood–brain-barrier penetrance and pharmacokinetic profile to show the transferability of the anti-prionic mode of action in vivo.

## Methods

### Recombinant expression and purification of monomeric wt and α-syn A140C

N-terminal acetylated α-syn wt (hereinafter referred to as α-syn) and acetylated α-syn-A140C were expressed in *E. coli* BL21(DE3) carrying codon-optimized α-syn in pT7 vector and the pNatB vector with the N-terminal acetylation enzyme from *Schizosaccharomyces pombe*^48^. Expression was performed in LB or ^15^N-supplemented M9-minimal medium with 1 mM IPTG after reaching an OD_600_ of 1.2, followed by incubation for 4 h at 37 °C. Purification was performed as described previously^49^ with some modifications: The pellets from 1 l expression were resuspended in 25 ml 20 mM Tris pH 8.0, and boiled at 95–100 °C for 2 × 15 min. After centrifugation at 20,000×*g* for 30 min at 4 °C, the supernatant was precipitated using a final concentration of 0.45 g/ml of ammonium sulfate. The protein was pelleted at 20,000×*g* for 30 min and resuspended in 50 ml 20 mM Tris–HCl pH 8.0. After sterile filtration, the sample was loaded on a HiPrep QFF 16/10 (Cytiva, USA, CV = 20 ml) anion exchange column. Gradient elution was performed with a target concentration of 800 mM NaCl over 20 CV. Recombinant α-syn eluted at a conductivity of 28–32 mS/cm. The fractions containing recombinant α-syn were pooled and precipitated using ammonium sulfate as described previously. The pellets were resuspended in 5 ml 50 mM Tris–HCl pH 7.4 50 mM NaCl, and loaded on a HiLoad Superdex 60/75 pg gel filtration column (Cytiva, USA, CV = 120 ml). The expression yielded 20–30 mg/l as determined by *A*_275_ with an extinction coefficient of 5600 M^−1^ cm^−1^. Protein aliquots were frozen with liquid nitrogen and stored at −80 °C.

### Mirror-image phage display selection

In phage display, exogenous peptides are presented on phage particles by fusion with the major coating proteins. Consecutive rounds of biopanning and amplification increase the fraction of phages presenting strong target binders, which is detectable by sequencing of the variable portion of the genome^50^. Using mirror-image phage display, the l-enantiomeric selection target is replaced by an otherwise identical d-enantiomeric version. This allows the identification of d-enantiomeric peptides that show high affinity for the physiological l-target and are more resistant to metabolic degradation than their l-enantiomeric counterparts^51,52^.

For mirror-image phage display, the commercially available M13-bacteriophage library TriCo-16 (Creative Biolabs, USA) was used. The library has a capacity of 2.6 × 10^10^ pIII fused 16-mer peptide variants. d-enantiomeric full-length α-syn carrying a C-terminally biotinylation and N-terminal acetylation was purchased as lyophilized powder from P&E (Peptides and Elephants, GE) with a purity of >90%. To minimize non-target related peptide enrichment, the display format was alternated between a polystyrene and polypropylene streptavidin functionalized 96-well plate surface (maximum capacity plates, BioTeZ, GE). For target immobilization, d-enantiomeric α-syn was diluted to a concentration of 2 pmol/well. Non-coupled streptavidin was quenched using biotin. The selection was performed as described previously^53–55^ with some minor modifications. Briefly, three consecutive selection rounds were performed using alternating blocking conditions with bovine serum albumin (BSA) and milk powder (MP) with PBS pH 7.4 as the selection buffer. Selection pressure was stepwise increased with each selection round using 5–10 washing repetitions. The selection was performed in three consecutive rounds on the target (target selection = TS). Additionally, input phages resulting from selection rounds on the target were incubated on a surface without target (direct control = DC), which was otherwise treated identically to the TS surface. As a second control, a consecutive selection was performed exclusively without a target on otherwise identically treated surfaces (empty selection = ES). A concentration of 2 × 10 ^12^ CFU ml^−1^ was used as input for all selection rounds and controls.

### Enrichment ELISA

Enrichment ELISA is a method to identify enrichment of target-binding phages during phage display selection. All steps were performed as described previously with some minor changes^53^. Briefly, 20 pmol/well of the d-enantiomeric α-syn target was immobilized on a streptavidin-coated polystyrene 96-well plate (maximum capacity plates, BioTeZ, GE). Both the target immobilized and the target-free surface were quenched with biotin. In total, 2.5 × 10^11^ phages from the TS input samples were diluted in 100 µl washing buffer and incubated on the target and control surface. The *A*_450_ of the product of the peroxidase reaction product 3,3’,5,5’-tetramethylbenzidine diimine was quantified after reaction stop with H_2_SO_4_ by absorption measurement in a Fluorostar Optima plate reader (BMG Labtech, GE; *n* = 3).

### ssDNA purification and next-generation sequencing of phage input samples

The ssDNA of the input phage suspensions resulting from mirror-image phage display selection was purified by phage precipitation and subsequent ssDNA separation as described previously^53–55^. For next-generation sequencing, PCR was performed, adding adapter sequences to both the 3’ and 5’ ends of the amplification product. Amplicon next-generation sequencing was performed by BMFZ-GTL Düsseldorf (GE) with a MiSeq system (Illumina, USA).

### Analysis of the next-generation sequences by filtering and clustering

Variable DNA sequences resulting from next-generation sequencing (NGS) were transcribed to peptide sequences as described previously^56^. The transcribed sequences were filtered based on their frequency increase in TS (library <TS1 < TS2 < TS3), their correlation with the presence of the target (TS2 > DC2; TS3 > DC3) and their frequency in the selection without target (TS1 > ES1; TS2 > ES2; TS3 > ES3). Sequences that passed the filter were ranked according to their enrichment from library to TS3 (TS3/library = enrichment score) and their frequency in TS3 compared to ES3 (TS3/ES3 = empty score)^56^. Filtered sequences were used as input for *Hammock clustering software*^57^. The input FASTA file included all filtered sequences as ranked by their *empty score*. *Hammock clustering software* was run in full mode with sequences ranked according to their input position (-R input). Cluster motif logos were generated from initial clusters after greedy clustering using the *WebLogo 3.4* application.

### d-enantiomeric peptides

d-enantiomeric peptides were purchased with C-terminal amidation from CASLO (CASLO, DK) as lyophilized chloride salt powder with a purity of >95%. SVD-1 and SVD-1a were tested in different buffer conditions, including PBS pH 7.4, where UV–vis absorption measurements after 1 h incubation at 37 °C and 20,800×*g* centrifugation showed that both compounds were completely retained in the supernatant up to at least 1 mM initial concentration.

### Thioflavin T assay

The Thioflavin T (ThT) assay is commonly used for the visualization of α-syn fibrilization, since the dye ThT is able to bind to the amyloidogenic cross-β-sheet proportions of fibril structures. Recombinant α-syn was thawed on ice and centrifuged for 30 min at 21,000 × *g* and 4 °C. The concentration of the supernatant was determined as described previously. Lyophilized d-peptides were thawed at RT for 1 h and dissolved in 500 µl PBS pH 7.4. After centrifugation for 30 min at 21,000 × *g* the supernatant concentration was determined by UV–vis using the corresponding extinction coefficient at *A*_280_. All ThT assay experiments were performed at 37 °C with 15 µM ThT and 0.05% sodium azide (w/v) in PBS pH 7.4 if not otherwise stated. ThT fluorescence was monitored with bottom optics at *λ*_ex_ = 448 nm and *λ*_em_ = 482 nm in a fluorescence plate reader with orbital averaging on 3 mm (Clariostar or Polarstar Optima, BMG labtech, GE). Prior to measurement start, 120 µl sample solution was transferred to a non-binding 96-half area well plate with transparent flat bottom (Corning, USA). One borosilicate glass bead (*d* = 3.0 mm, Hilgenberg, GE) was added to each well for all de novo aggregation assays. For seeded aggregation assays, no bead was used, and samples were incubated under quiescent conditions. Wells that surrounded the sample wells were filled with the same volume of buffer to improve heat distribution. Experiments were performed with five replicates (*n* = 5) if not otherwise stated.

ThT assay was used for different purposes. First, de novo ThT aggregation assays served as a screening platform for the aggregation delay with the synthetic D-peptides. Here, 50 µM recombinant α-syn was incubated with a three-fold molar excess of each d-peptide, respectively. Samples were shaken before each cycle using orbital shaking mode at 300 rpm for 30 s. Peptides that were insoluble in aqueous buffer were dissolved in 2.5 µl DMSO (0.5 mg peptide) and gradually mixed with PBS pH 7.4 until a final concentration of 2.5% (v/v) DMSO was reached. For these samples, the reference aggregation of α-syn alone was also performed in PBS pH 7.4 with 2.5% (v/v) DMSO. Aggregation curves of each replicate were individually fitted using a symmetric Boltzmann sigmoidal fit (OriginPro 2020, OriginLab, USA) with the following formula: $y=\,\frac{{A}_{1}-\,{A}_{2}}{1+{{\rm{e}}}^{(x-{x}_{0})/{\rm{d}}x}}+{A}_{2}$ (*A*_1_ = initial value, *A*_2_ = final value, *x*_0_ = inflection point [s], d*x* = time constant [1/s]). The inflection point of the fit determines the aggregation half-time *t*½, whereas the lag-time was approximated with the following formula: *t*_1/2_–2 × d*t*_1/2_, where d*t*_1/2_ is defined as the slope of the fit at *x* = *t*_1/2_ in 1/s^58^_._ Half-time and lag-time were calculated as the mean value of the separate fits. For the concentration dependency of aggregation delay, different compound concentrations were applied to 50 µM recombinant α-syn. Lag-time and *t*½ were calculated as described previously. For graphical representation, the fitted steady-states were normalized to 1 for all conditions and the mean error was calculated based on the fits. Statistical testing on the significance of the time shifts was performed using the two-sample Welch’s *t*-test with *p* < 0.05 (OriginPro 2020, OriginLab, USA). For de novo ThT assays including sub-stoichiometric compound concentrations, 10 µM α-syn was used. In contrast to the screening aggregation assay, α-syn samples were continuously shaken at 300 rpm using orbital shaking mode to reduce the aggregation time, taking measurements every 5 min. Statistical evaluation was performed as described previously by comparing the significance of *t*½, lag-time shifts and steady state reduction in the presence of the inhibitor and the same concentration of the corresponding control peptide. For seeded ThT assays, 50 nM monomer equivalent small α-syn PFF seeds were incubated ON at 37 °C under quiescent conditions together with or without different concentrations of the compounds in the fluorescence plate reader. After 20 h 20 µM monomeric α-syn was added to the sample mixture to start seeding. Measurements were taken every 5 min (*n* = 3).

### *Preparation of* small α-syn PFF seeds

small α-syn PFF seeds were prepared as described previously with some modifications^21^. First, insoluble PFF were produced by incubation of 300 µM recombinant α-syn in a LoBind reaction tube (Eppendorf GmbH, GE) with one borosilicate glass bead (*d* = 3.0 mm; Hilgenberg, DE) in 20 mM NaPi, pH 7.0, 150 mM NaCl, 0.05% (w/v) sodium azide for one week at 37 °C. The insoluble PFF were harvested by ultracentrifugation at 100,000 × *g* for 30 min at 4 °C, and the pellet was washed several times with 20 mM NaPi, pH 7.0, 150 mM NaCl. The monomer equivalent concentration was determined by measuring the α-syn concentration in the supernatant after the first centrifugation and subtracting it from the start concentration for fibrilization. The insoluble PFF were resuspended in buffer and frozen at −80 °C with liquid N_2_. Small α-syn PFF seeds were generated by harsh sonication of 200 µl insoluble PFF with 300 µM monomer equivalent concentration for 3 × 15 s (1 s on/off) and 60% amplitude with a tip sonifier (MS 72 micro tip, Sonopolus, Brandelin, GE). Insoluble PFF were separated by centrifugation at 100,000×*g* for 1 h at 4 °C. The supernatant containing small α-syn PFF seeds was separated, aliquoted and frozen at −80 °C with liquid N_2_.

### Surface plasmon resonance kinetic experiments

Measurements were performed using an 8K Biacore device (Cytiva, USA). Interactions were measured using single-cycle kinetics experiments. For all assays, the peptide compounds were immobilized as ligand on the sensor surface and recombinant α-syn was injected as analyte in the flow. SVD-1 and SVD-1a were immobilized via primary amino groups on a CMD200M carboxyldextran matrix chip (Xantec, GE). Immobilization was performed after 7 min EDC/NHS activation at 10 µl/min with 50 µg/ml peptide in 10 mM NaAc, pH 5.0 for SVD-1 and pH 7.0 for SVD-1a until a saturation signal was reached (SVD-1: 400 RU, SVD-1a: 500 RU). Surface quenching was performed using 1 M ethanolamine, pH 8.3. The kinetic experiments were performed using a flow rate of 30 µl/min in PBS pH 7.4 if not otherwise stated. The surface was regenerated in between cycles using 30 s injections of 2 M Gua-HCl at 30 µl/min. Data evaluation was performed using Biacore *Insight Evaluation Software* v3.0 (Cytiva, USA).

### SVD-1a_Cys_MTSL spin label preparation

The spin-labeled analog of SVD-1a was prepared by covalent attachment of MTSL (S-(1-oxyl-2,2,5,5-tetramethyl-2,5-dihydro-1H-pyrrol-3-yl)methyl methanesulfonothioate, Toronto Research Chemicals, USA) to the C-terminal D-cysteine residue of SVD-1a_Cys. MTSL was dissolved in DMF (N,N-dimethylformamide) with a concentration of 20 mM and diluted in 200 mM HEPES, pH 7.6, to a final concentration of 2 mM. 900 µl of the solution was then added to 1 mg of lyophilized SVD-1a_Cys to create a fivefold molar excess of MTSL compared to SVD-1a_Cys. The reaction mixture was incubated for 2 h at RT and subsequently applied to a semipreparative RP-HPLC C8 column (Zorbax-300 SB, Agilent, GE) connected to an HPLC system (Agilent 1260, Agilent, GE). Purification of the spin-labeled peptide SVD-1a_Cys_MTSL was achieved by applying an aqueous acetonitrile (ACN) gradient (8% ACN, 0.1% trifluoroacetic acid (TFA) to 60% ACN, 0.1% TFA in Milli-Q water within 40 min), running at a flow rate of 4 ml min^−1^ at 25 °C with a detection at 214 nm. The purified reaction product was flash-frozen with liquid N_2_ and lyophilized (LT-105, Martin Christ, GE). The purity of the SVD-1a_Cys_MTSL spin-labeled peptide was verified by RP-HPLC with >98%.

### NMR spectroscopy

Samples were prepared at final concentrations of 25 µM ^15^N-labeled full-length acetyl-α-syn in the absence (reference) and presence of an equimolar amount of SVD-1a (not isotopically labeled, thus NMR-invisible) in PBS buffer, pH 7.4, with an addition of 5% D_2_O for internal reference. 2D 1H-15N HSQC spectra were recorded back-to-back, on a Bruker AVANCE NEO spectrometer (Bruker, USA) operating at 1200 MHz proton Larmor frequency. The experimental temperature was 10 °C. Spectral dimensions were 16.02 ppm (^1^H) x 30 ppm (^15^N), with 2048 points in the ^1^H dimension and 256 increments in the ^15^N dimension, resulting in an acquisition time of 53 ms for the ^1^H dimension and 35 ms for the ^15^N dimension. For each increment, 32 scans were recorded, with a recovery delay of 1 s between scans, resulting in an overall experimental time of 4.8 h per spectrum.

NMR paramagnetic relaxation enhancement (PRE) data were recorded on 25 µM ^15^N-labeled full-length acetyl-α-syn in the presence of 25 µM paramagnetically labeled (but not isotopically enriched) SVD-1a_Cys_MTSL (using a MTSL spin-label covalently attached to the SVD-1a C-terminus), resulting in a 1:1 ratio of α-syn: SVD-1a. Intensities (*I*_para_) were extracted from 2D ^1^H–^15^N Best-TROSY NMR spectra recorded at 600 MHz and 10 °C, each with 128 scans per increment, resulting in a total experimental time of 16 h per spectrum. Reference data were obtained by adding a 20-fold molar excess of ascorbic acid to the same sample, therefore quenching the paramagnetic effect of the spin-label and obtaining a diamagnetic reference sample. The diamagnetic reference spectra and intensities (*I*_dia_) were recorded back-to-back and under identical conditions as for the paramagnetic sample.

NMR data sets were processed using the Bruker TopSpin software (version 4.1.1) and visualized using *CcpNmr Analysis* (v2.4.2)^59^. For the assessment of chemical shift changes of α-syn resonances in the presence of SVD-1a relative to the reference spectrum (without SVD-1a), peak positions were extracted using the *CcpNmr Analysis* software. From the residue-specific chemical shift changes in the ^1^H and ^15^N dimensions, an absolute chemical shift change, Δ*δ*, was calculated using the formula Δ*δ* = $\sqrt{0.5\left({\delta }_{{\rm{H}}}^{2}+\left(0.14\,{\delta }_{{\rm{N}}}^{2}\right)\,\right)}$ ^60^. For analysis of the PRE data, resonance intensities of the paramagnetic sample and the diamagnetic reference sample peak intensities were extracted using *CcpNmr analysis*.

### Dynamic light scattering (DLS)

Measurements were performed using a SpectroSize 300 131 (XtalConcepts, GE) instrument and a sample volume of 1 ml in a sealed quartz cuvette (Hellma Group, GE). Samples were incubated in PBS pH 7.4 at 37 °C under quiescent conditions. Prior to measurements, all samples were centrifuged at 21,000×*g* for 30 min at 4 °C to remove potential impurities from the solution. For time-dependent DLS measurements, data points were recorded every 60 s. Diffusion coefficients were obtained from analysis of the decay of the scattered intensity autocorrelation function and were used to determine apparent hydrodynamic radii via the Stokes-Einstein equation. Kinetic fitting for the degradation of small α-syn PFF seeds was performed with the “SVD-1a + small α-syn PFF” sample using the “single-phase exponential decay function” of GraphPad Prism 10 (GraphPad Software Inc., USA), considering only particle sizes in the range of 1–100 nm with an amplitude > 0.2. Outliers were identified with a *Q* value of 1% and excluded from the fitting. Protein quantification after centrifugation was performed by incubating identical samples as in the DLS measurements with additional compound and buffer controls in low-binding reaction tubes at 37 °C under quiescent conditions (*n* = 3). After 0, 20 and 72 h of incubation, the samples were centrifuged at 21,000×*g* for 30 min at RT, and the supernatant was carefully transferred to a fresh reaction tube, while potential pellets were resuspended in 1 ml PBS 7.4. Protein quantification of pellet and supernatant was performed using a Micro-BCA kit following the manufacturer’s instructions (Thermo Fisher Scientific, USA).

### Western blot

Samples of 200 nM small α-syn PFF or α-syn monomer were incubated with 0, 250, 500, 750 and 1000 nM SVD-1a for 72 h at 37 °C in PBS pH 7.4 with 0.05% sodium azide. Samples were then resolved via SDS–PAGE on 12% TGX gels (Bio-Rad, Hercules, USA) using the Mini-PROTEAN Tetra Cell (Bio-Rad, Hercules, USA). Proteins were transferred to a PVDF membrane (0.2 µm pore size) (Trans-Blot Turbo Mini PVDF Transfer Pack, Bio-Rad, Hercules, USA) at 25 V, 1.3 A for 7 min. Blocking of the membrane was carried out with 2% nonfat dried milk powder in TBS + 0.05% Tween 20 for 1 h at room temperature. Anti-α-synuclein antibody Syn211 (RRID:AB_628318, Santa Cruz Biotechnology, Dallas, TX, USA) was used as primary antibody at a concentration of 1 µg/ml in TBS-T for 1 h at RT. As a secondary antibody, an HRP-coupled goat anti-mouse IgG (RRID:AB_228307, Thermo Fisher Scientific, Waltham, MA, USA) was used at 0.2 µg/ml in TBS-T for 1 h at RT. The membrane was incubated with Pierce™ ECL Western Blotting substrate (Thermo Fisher Scientific, Waltham, MA, USA) for 1 min, and protein bands were visualized with a UVP ChemStudio (Analytik Jena, Jena, Germany).

### α-Syn size-based fractionation by centrifugal concentration followed by ELISA quantification or SDS PAGE analysis

For small PFF pre incubation with or without SVD-1a (Fig. 4) samples were pre-incubated at 37 °C and 600 rpm for 72 h in PBS pH 7.4 with 0.05% sodium azide using protein low binding reaction tubes in a thermos shaker (Eppendorf GmbH, GE). For de novo aggregation analysis, each replicate was pre-incubated in PBS pH 7.4 with 0.05% sodium azide in a 96-well non-binding half-area plate (Corning, USA) in a FLUOStar platereader (BMG labtech, GE) with 300 rpm continuous orbital shaking in between reads, adding one borosilicate bead per well (*d* = 3 mm, Hilgenberg, GE, *n* = 7). 10 µM α-syn monomer was incubated with or without 5, 10 or 20 µM SVD-1a for 7 days. Replicates were united and 500 µl (PFF experiment) or 200 µl (de novo aggregation) of each condition was loaded on an equilibrated centrifugal concentrator with a MWCO of 100 kDa (Merck, Microcon DNA Fast Flow 100 MWCO, GE) and centrifuged for 30 min at 500×*g* at RT. The volumes of retentate and flow through were adjusted with buffer to the initially loaded volume. The α-syn concentration of the samples was then quantified using an α-syn-specific kit (Human α-Synuclein (Colorimetric), 448607, BioLegend, USA). For de novo aggregation analysis by SDS–PAGE the samples were supplemented with Laemmli buffer (375 mM Tris–HCl (pH 6.8), 9% SDS, 50% glycerol, 9% β-mercaptoethanol, 0.03% bromophenol blue) and boiled for 5 min. 15 µl of each sample and 8 µl of protein standard (Nove Sharp Pre-stained Protein Standard, Thermo Fisher Scientific, USA) were applied to a 15% SDS gel. Staining was performed using Pierce Silver Stain Kit following the manufacturer’s instructions (Thermo Fisher Scientific, USA).

### Atomic force microscopy (AFM)

Samples were prepared by dilution to 1 µM α-syn monomer concentration, and 5 µl was incubated and dried on a freshly cleaved mica surface. Surfaces were then three-times washed with 200 µl ddH_2_O and dried using a gentle stream of N_2_. Measurements were performed in a Nanowizard 3 system (JPK BioAFM—Bruker Nano GmbH, GE) using intermittent contact mode with 2 × 2 and 5 × 5 µm section and line rates of 0.5–2 Hz in ambient conditions using a silicon cantilever and tip with a nominal spring constant of 26 N/m, average tip radius of 9 ± 2 nm and a resonance frequency of ~300 kHz (Olympus OMCL-AC160TS-R3). The images were processed using JPK data processing software (version spm-5.0.84). For the height profiles presented, a polynomial fit was subtracted from each scan line, first independently and then using a limited data range.

### Circular dichroism (CD) spectroscopy

Far-UV circular dichroism (CD) data were collected using a Jasco J-1100 spectropolarimeter (Jasco, GE). 350 µl samples were pooled and loaded into a high precision quartz cuvette with a path length of 1 mm (Hellma group, GE). A scan speed of 20 nm/min with five accumulations per sample was performed using far UV wavelengths from 260 to 190 nm. Baseline was corrected by subtracting measurements of the reference sample only.

### Cell assay for α-synuclein aggregation

A construct encoding full-length A53T-mutated human α-syn fused with YFP at the C-terminus was synthesized and introduced into the pMK–RQ expression vector (GeneArt; Thermo Fisher Scientific, USA). The α-synA53T–YFP construct was subcloned into the pIRESpuro3 vector (Clontech; Takara Bio, JPN) using NheI (5′) and NotI (3′) restriction sites. HEK293T cells (American Type Culture Collection) were cultured in high-glucose Dulbecco’s modified Eagle’s medium (DMEM; Sigma-Aldrich, USA) supplemented with 10% fetal calf serum (Sigma-Aldrich, USA), and 50 units/ml penicillin as well as 50 μg/ml streptomycin (Sigma-Aldrich, USA). Cells were cultured in a humidified atmosphere of 5% CO_2_ at 37 °C. Cells plated in DMEM were transfected using Lipofectamine 2000 (Invitrogen; Thermo Fisher Scientific, USA). Stable cells were selected in DMEM containing 1 μg/ml puromycin (EMD Millipore, USA). Monoclonal lines were generated by fluorescence-activated cell sorting of a polyclonal cell population in 96-well plates using a MoFlo XDP cell sorter (Beckman Coulter, USA). Finally, the clonal cell line B5 was selected from among 24 clonal cell lines and is referred to as αSynA53T–YFP cells. Peptides were incubated with 1.5% Lipofectamine 2000 in OptiMEM for 2 h at room temperature. The α-synA53T–YFP cells were plated in a 384-well plate with poly-D-lysine coating (Greiner, AT) at a density of 1000 cells per well with 0.1 μg/ml Hoechst 33342 (Thermo Fisher Scientific, USA), and the previously prepared transfection mix was added directly to the cells in the well. To seed cellular aggregation of α-syn in α-synA53T–YFP cells, 30 nM soluble α-syn small α-syn PFF seeds were incubated with 1.5% Lipofectamine in OptiMEM for 2 h at room temperature and then added to each well 3 h after the first transfection. The plate was then incubated in a humidified atmosphere of 5% CO_2_ at 37 °C. On day 3 the cells were imaged with an IN Cell Analyzer 6500HS System (Cytiva, USA) using the blue and green fluorescence channel, and analyzed using IN Carta Image Analysis Software (Cytiva, USA) after an algorithm was established to identify intracellular aggregates in living cells. For each condition, we used four wells and took 16 images per well, which were analyzed by a fully automated algorithm to avoid bias. Statistical analysis was performed using one-way ANOVA followed by Dunnett’s multiple comparisons test (GraphPad Prism 9, GraphPad Software, USA). Error bars represent standard deviation.

### Cell-viability assay (CellGlo test)

We used the CellTiter-Glo Luminescent Cell Viability Assay (Promega GmbH, GE) to determine the number of viable cells in culture based on quantitation of the ATP present, an indicator of metabolically active cells. After culturing cells in 384-well plates for three days, 35 µl of medium was removed from the wells and 40 µl of CellTiter-Glo Reagent directly added to each well. After mixing, luminescence was measured 10 min later using a FLUOStar (BMG labtech, GE).

### Immunofluorescent cell staining

After culturing cells for 3 days on 384-well plates, the cells were fixed in 4% formaldehyde (Sigma-Aldrich, USA) in PBS (pH 7.4) for 15 min. After washing three times with PBS for 5 min each, the cells were permeabilized with 0.25% Triton X-100 (Sigma-Aldrich, USA) in PBS for 10 min. After another three washes with PBS for 5 min each, the cells were blocked with 1% bovine serum albumin (Sigma-Aldrich, USA) in PBS supplemented with 0.1% Tween 20 (Sigma-Aldrich, USA) for 30 min. The cells were stained with CF633 (Biotium, USA) fluorescently labeled antibodies at 8 µg/ml in 1% bovine serum albumin in PBS supplemented with 0.1% Tween-20 for 1–3 h at room temperature in the dark. For detecting total α-syn, we used the anti-α-syn antibody syn211 (Abcam, UK). For detecting oligomeric and fibrillar α-syn, we used the anti-aggregated α-syn antibody, clone 5G4 (Sigma-Aldrich, USA). For detecting α-syn phosphorylated at serine 129, we used the recombinant anti-α-syn (phospho S129) antibody EP1536Y (Abcam, UK). After a final three washes in PBS for 5 min each, the cells were imaged in PBS using an IN-Cell Analyzer 6500HS System and 40-fold magnification (Cytiva, USA).

### Cell viability assay (MTT test)

The potential cell viability rescue of PC12 cells (Leibniz Institute DSMZ, GE) from α-syn toxicity through the addition of SVD-1, SVD-1a or SVD-1_scrambled was measured in an MTT (3-(4,5-dimethylthiazol-2-yl)-2,5-diphenyltetrazolium bromide) cell viability test. PC12 cells (Leibniz Institute DSMZ, GE) were cultivated on collagen A-coated (Biochrom GmbH, GE) tissue culture flasks in RPMI 1640 medium supplemented with 5% fetal calf serum and 10% horse serum in a 95% humidified atmosphere with 5% CO_2_ at 37 °C. 10,000 cells per well in a volume of 100 µl were seeded on collagen A-coated 96-well plates (Thermo Fisher Scientific, USA) and were incubated for 24 h at 37 °C and 300 rpm in a thermo cycler. Then, final concentrations of 30 nM α-syn either in the absence or after pre-incubation with 15 µM SVD-1, SVD-1_scrambled or 0.5 µM SVD-1a were added to the cells. In addition, 15 µM of the peptides alone, cell media, buffer without peptides and 0.1% Triton X-100 (cytotoxic compound) served as controls. After further incubation in a 95% humidified atmosphere with 5% CO_2_ at 37 °C for 24 h, cell viability was measured using the Cell Proliferation Kit I (MTT) (Roche Applied Science, CH) according to the manufacturer’s protocol. The MTT formazan product was quantified by measuring the absorbance at 570 nm corrected by subtraction of the absorbance at 660 nm in a FLUOStar Optima plate reader (BMG Labtech, GE). All results were normalized to untreated cells grown in medium only. Test on significance was performed using one-way ANOVA with Bonferroni post hoc analysis (OriginPro 2020, OriginLab, USA; *n* = 4).

## Supplementary information

Supplementary Files combined

## Data Availability

The datasets generated during and/or analyzed during the current study are available from the corresponding author on reasonable request.
