# Supplementary material for: Direct disassembly of α-syn preformed fibrils into α-syn monomers by an all-D-peptide
Source: NPJ Parkinsons Dis. 2025 Sep 22;11:271. doi: 10.1038/s41531-025-01132-7 (PMC12454639; doi:10.1038/s41531-025-01132-7)
Supplement: Supplementary file 1 — Supplementary Files combined [file 41531_2025_1132_MOESM1_ESM.docx]

**SUPPLEMENTARY INFORMATION**


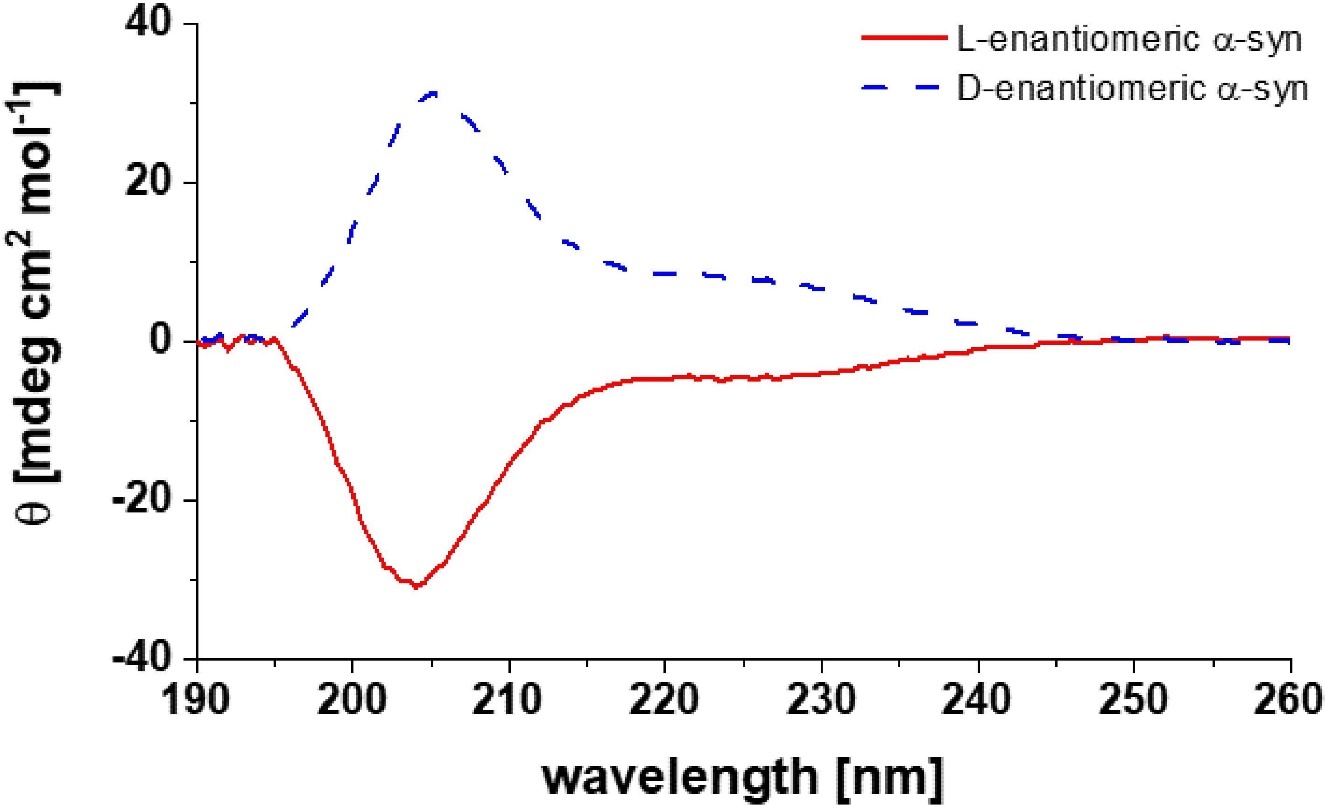


**Supplementary Figure 1: CD-measurement of recombinant l- and synthetic d-enantiomeric full-length α-syn**. The d-enantiomeric C-terminally biotinylated synthetic α-syn was used as selection target. Measurements of 20 µg ml^-1^ protein in 50 mM NaPi pH 7.4 were performed as described in the method section.


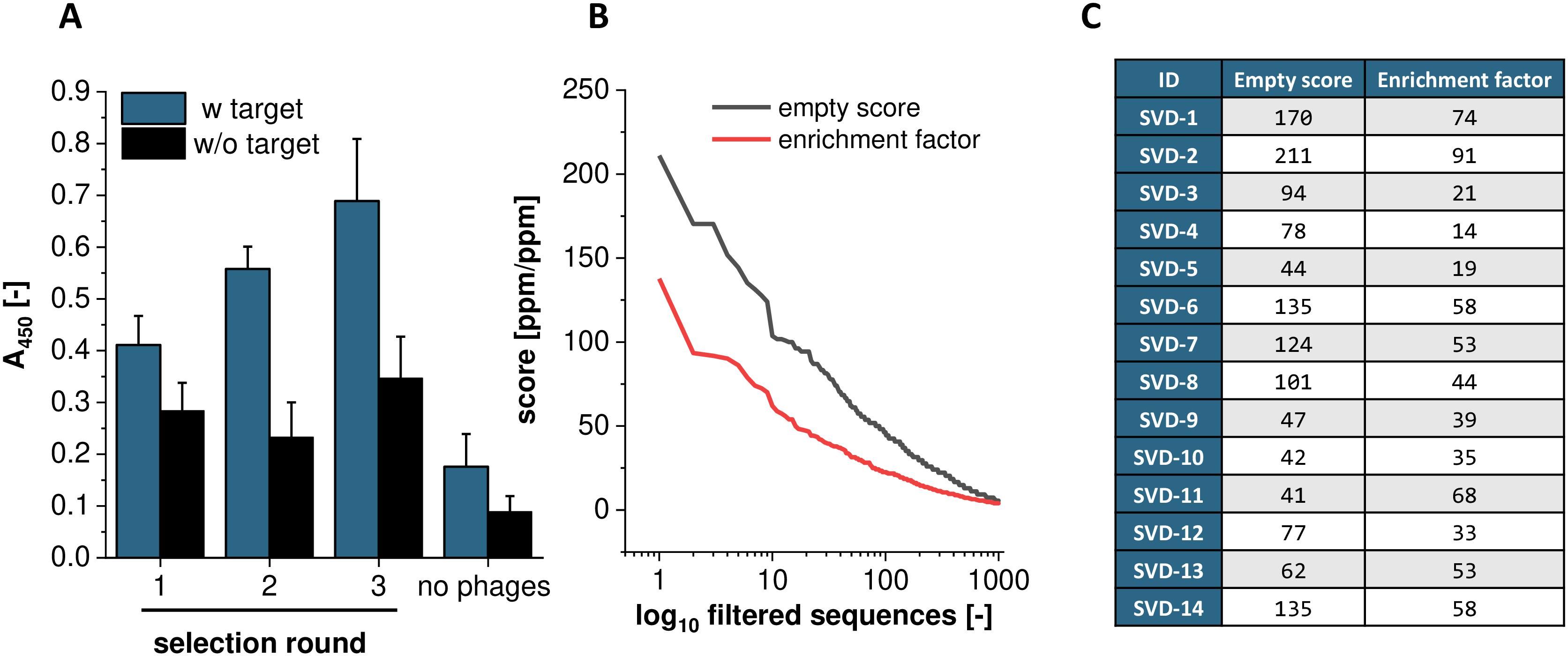


**Supplementary Figure 2: Results of the mirror image phage display selection and NGS analysis**. Mirror-image phage display and NGS analysis was performed as described as follows: The full-length D-enantiomeric α-syn target protein (Supplementary Fig. 1) was immobilized by directed coupling on a streptavidin derivatized surface via a C-terminal biotin to ensure a homogenous interaction surface during biopanning with the 16-mer M13 phage library. In addition to incubation of the phage library with the target protein (TS = target selection), an identical parallel selection, which lacks the target immobilization (ES = empty selection), was performed. Each purified phage suspension resulting from one TS round was additionally incubated on a surface without target (DC = direct control). Enrichment of target binding phages was verified after three selection rounds by direct detection of the phage coating protein in an ELISA set-up. The purified DNA of the phage suspensions was analyzed by NGS resulting in 0.9 – 1.1 Mio total sequences per sample. Target related sequences were identified after normalization by filtering specifically for those sequences whose number increased during selection rounds (TS3 > TS2 > TS1 > library) and occurred with a higher frequency in TS than in the corresponding controls that miss the target (TS1 > ES1, TS2 >ES2, TS3 > ES3, TS2 > DC2, TS3 > DC3). Thus, the sequence pool was narrowed down to 140,000 sequence variants with ~ 4,000 showing more than one appearance. The sequences were ranked by a scoring system based on enrichment (enrichment factor = TS3/ library) and target related occurrence (empty score = TS3/ ES3), which allowed the identification of the sequence variants with the highest probability for a target interaction. In addition, clustering of the filtered sequences allowed the identification of motifs and possible cluster extensions. Based on these results, 14 d-enantiomeric synthetic peptides were further analyzed in in vitro experiments. (**A**) Enrichment ELISA of phage suspensions after amplification. 2.5 ∙ 10^11^ phages were incubated with or without 20 pmol/well immobilized d-enantiomeric full-length α-syn target protein (n = 3). (**B**) Scoring values for enrichment (empty score = TS3/ ES3) and target specificity (enrichment factor = TS3/ library) of the filtered sequences resulting from NGS sample analysis. (**C**) List of sequences that were selected for synthesis based on NGS-scoring, empty score and enrichment factor. These sequences were screened for aggregation delaying effects in ThT-assay as shown in Supplementary Fig. 4.


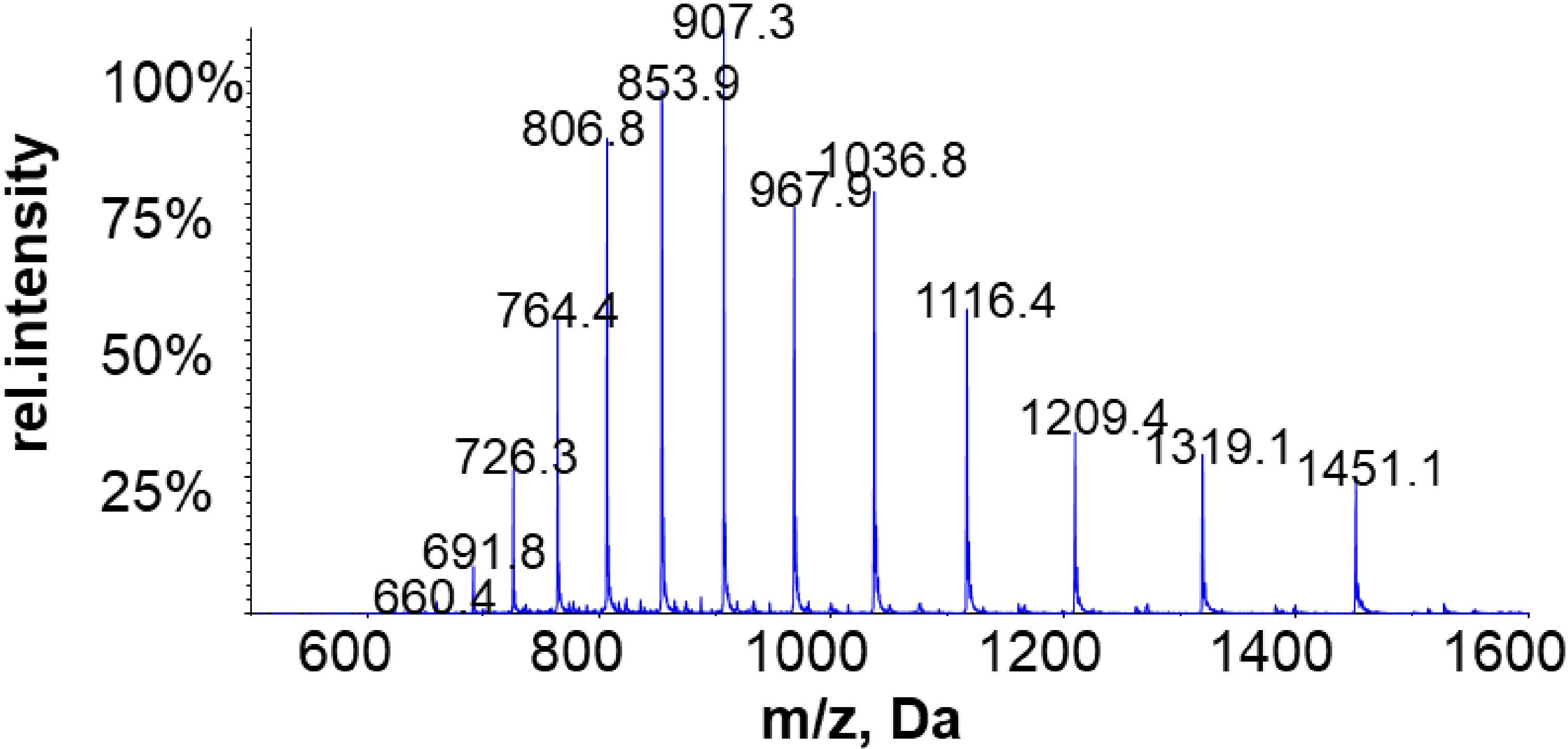


**Supplementary Figure 3: Mass spectrometry spectrum of recombinant α-syn.** An HPLC-MS method with ESI as an ionization mode with a Triple Quadrupole Qtrap6500 instrument (ABSciex, GE) coupled with an Agilent 1260 HPLC system (Agilent, GE) was used. The reversed-phase column Thermo, Accucore-150-C4 (100*4.6 mm 2.6 µm) was used with the following gradient: the gradient started with 5 % B for 5 min, followed by a 15 min ramp from 5 % to 9 % B followed by a hold of 95 % B for 5 min and a re-equilibration of 10 min with 5 % B. [(A: 0.1 % v/v formic acid in H_2_O), (B: 0.1 % v/v formic acid in ACN)] at 30°C and an injection volume of 10 µl. Mass deconvolution was performed with ESIProt ^58^).


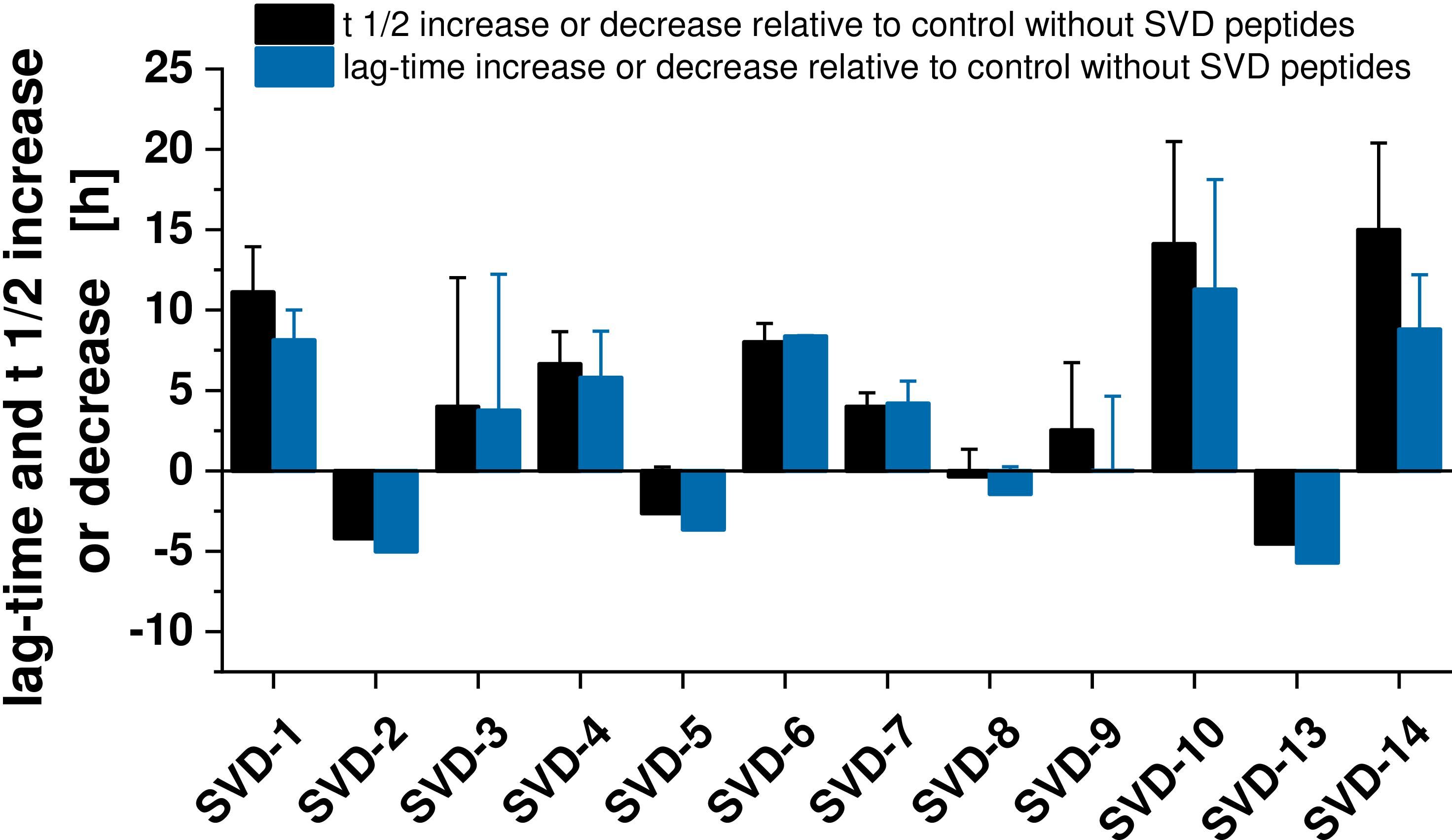


**Supplementary Figure 4: Thioflavin-T assay with α-syn and d-enantiomeric 16-mer peptides SVD-1 to SVD-14**. Monomeric α-syn (Supplementary Fig. 3) was incubated with or without a 3-fold molar excess of the D-peptides. Peptides SVD-11 and -12 were found to be insoluble in aqueous buffer and organic solvents and were therefore excluded from further analysis. The ThT positive aggregation was fitted with a symmetric Boltzmann fit and t ½- and lag-time were calculated accordingly. Since ThT signal is not always correlating with absolute amyloid mass (since different inhibitors can induce the formation of different fibril polymorphs) we decided not to evaluate the steady state in the early screening phase but rather concentrate on peptide induced t ½ - and lag-time shifts. 50 µM recombinant α-syn was incubated with or without a three-fold molar excess of the respective d-peptide in PBS pH 7.4 at 37°C. ThT fluorescence progression was measured in a 96-well non-binding half-area with a FLUOStar plate reader at λex = 448 nm and λem = 482 nm. Samples were shaken at 300 rpm for 30 s per cycle using orbital shaking mode t ½- and lag-time increase or decrease were calculated after Boltzmann sigmoidal fitting. The t ½ is given by the inflection point of the fit and the lag-time was approximated using the formula [lag‑time = t ½ - 2dx] where dx is defined as the slope of the fit at x = t ½. t ½ and lag-time increase or decrease were determined based on the differences between samples with and without SVD peptide, whereas the 0-value represents the reference control without inhibitor. Mean data are shown with ±SD (n = 3). For α-syn without peptide the following t ½- and lag-times were determined: t ½ 50.5 h (PBS) and 50.1 h (PBS + 2.5 % DMSO); lag-time: 38.2 h (PBS) and 38.9 h (PBS + 2.5 % DMSO). Non-normalized aggregation curves are provided in Supplementary Fig. 5. Seven out of twelve D-peptides delayed α-syn aggregation (SVD- 1, 3, 4, 6, 7, 10, 14), whereas three D-peptides promoted the aggregation onset (SVD 2, -5, -13) and two D-peptides did not show significant impact on the aggregation (SVD 8 and -9). Among the peptides that delayed the aggregation, SVD-1, -4, -6, -10 and -14 showed the strongest inhibitory effects ranging from + 5 to 15 h t ½ - and lag-time shifts. Next, to verify the concentration dependency of the inhibitory effect, α-syn was incubated with increasing peptide concentrations, starting with equimolar concentration and continuing with a three-fold and five-fold molar excess, respectively (Supplementary Fig. 6).


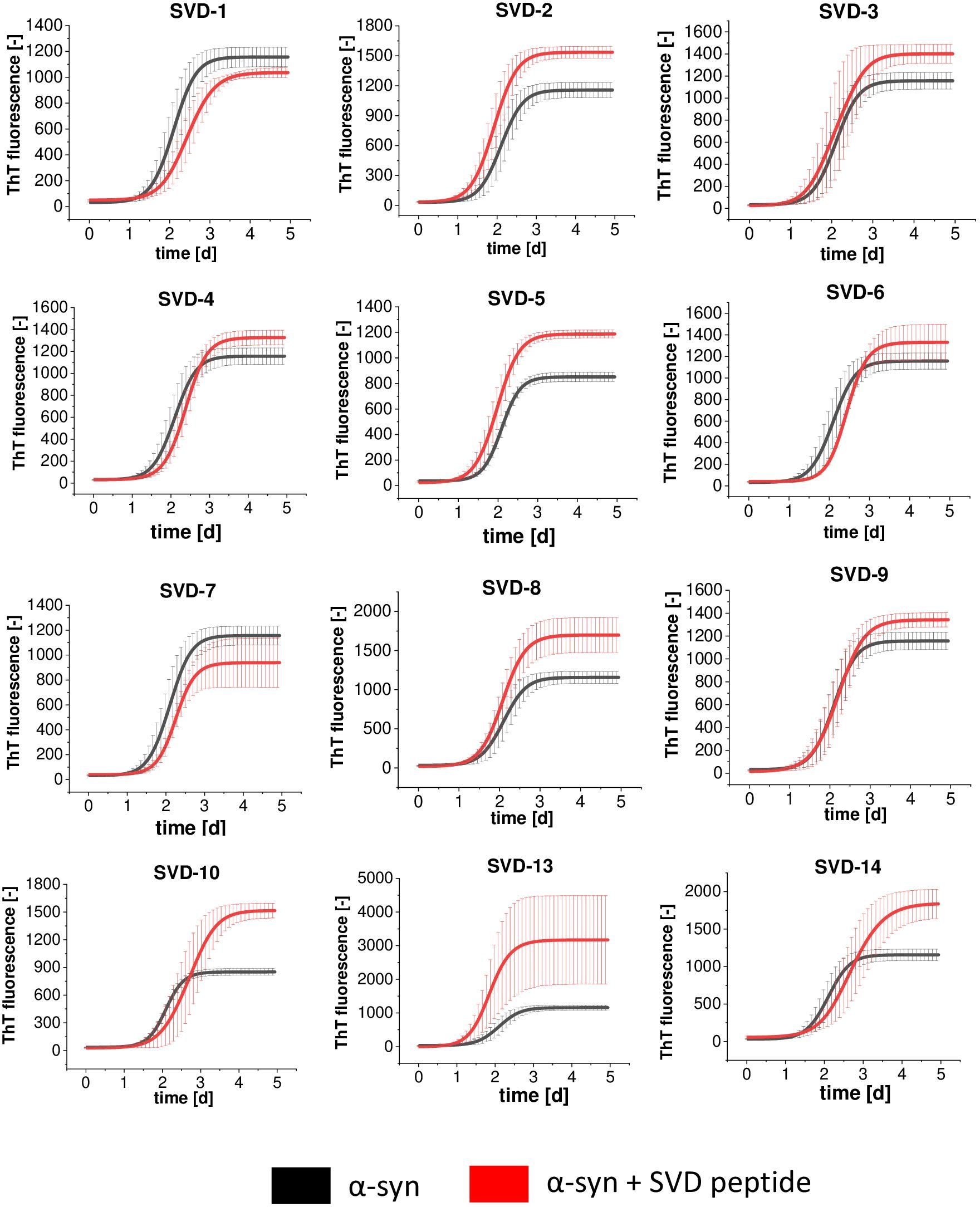


**Supplementary Figure 5:** **Non-normalized ThT data of *de novo* aggregation assay screen with α-syn and SVD peptides 1 to 14 as shown in Supplementary Fig. 4.** 50 µM recombinant full-length l-α-syn was incubated with or without a three-fold molar excess of the respective d-peptide in PBS pH 7.4 at 37°C. ThT fluorescence progression was measured in a 96-well non-binding half-area with a FLUOStar plate reader at at λex = 448 nm and λem = 482 nm. Samples were shaken at 300 rpm for 30 s per cycle using orbital shaking mode t ½- and lag-time shifts were calculated after Boltzmann sigmoidal fitting. Mean data are shown with ±SD (n = 3). For α-syn without peptide the following t ½- and lag-times were determined: t ½ 50.5 h (PBS) and 50.1 h (PBS + 2.5 % DMSO); lag-time: 38.2 h (PBS) and 38.9 h (PBS + 2.5 % DMSO).


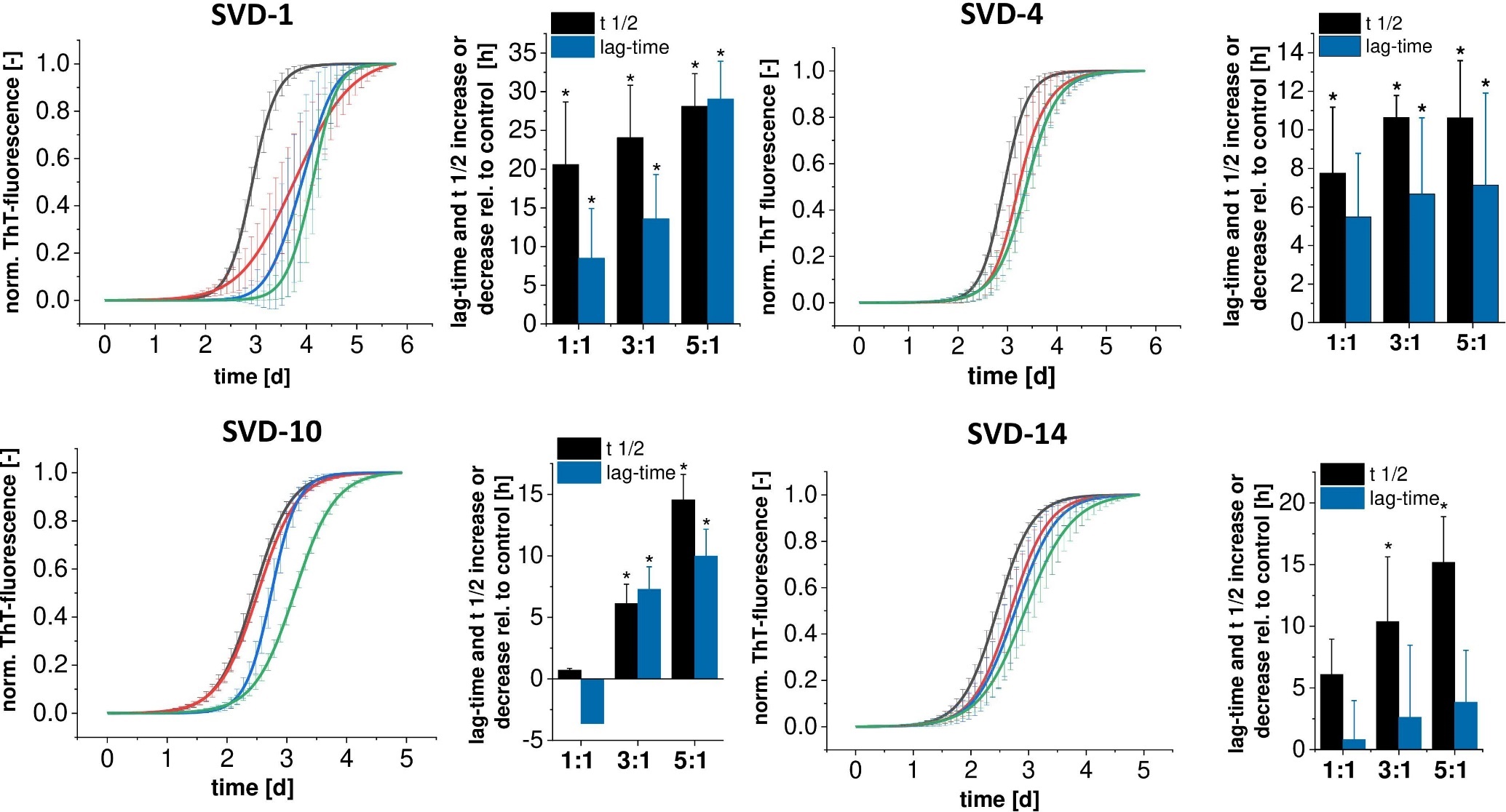


**Supplementary Figure 6: Thioflavin-T assay with α-syn and d-enantiomeric 16-mer peptides**. 50 µM α-syn was incubated with or without 50, 150 or 250 µM d-peptide in PBS pH 7.4 (black: 0 µM; red: 50 µM; blue: 150 µM; green: 250 µM). ThT fluorescence progression was measured in a 96-well non-binding half-area plate (Corning, USA) with a FLUOStar platereader (BMG labtech, GE) at λex = 448 nm and λem = 482 nm. Samples were shaken at 300 rpm for 30 s per cycle using orbital shaking mode. The five replicates of each condition were individually fitted with a Boltzmann fit. The t ½ is given by the inflection point of the fit and the lag-time was approximated using the formula [lag-time = t ½ - 2dx] where dx is defined as the slope of the fit at x = t ½. t ½ and lag-time increase or decrease were determined based on the differences between samples with and without peptide, whereas the 0-value the reference control without inhibitor. The significance of time-shifts was tested with a two sample Welch‘s t-test with p < 0.05 (significant: *). Mean data shown with ± SD (n = 5). For α-syn without peptide the following t ½- and lag-times were determined: For SVD-1 and SVD-4: t ½: 70.4 h and lag-time 58.9 h for SVD-10 and SVD-14: t ½: 59.8 h and lag-time 49.4 h. All of the tested D-peptides decelerated α-syn aggregation, with SVD-1 being most efficient, with a t ½-time shift of + 28 h ± 4.2 h and a lag-time shift of + 29 h ± 4.9 h for a five-fold molar excess. Because of these results, we decided to continue with SVD-1 for further verification of the sequence specificity of the observed effects (Fig. 2).


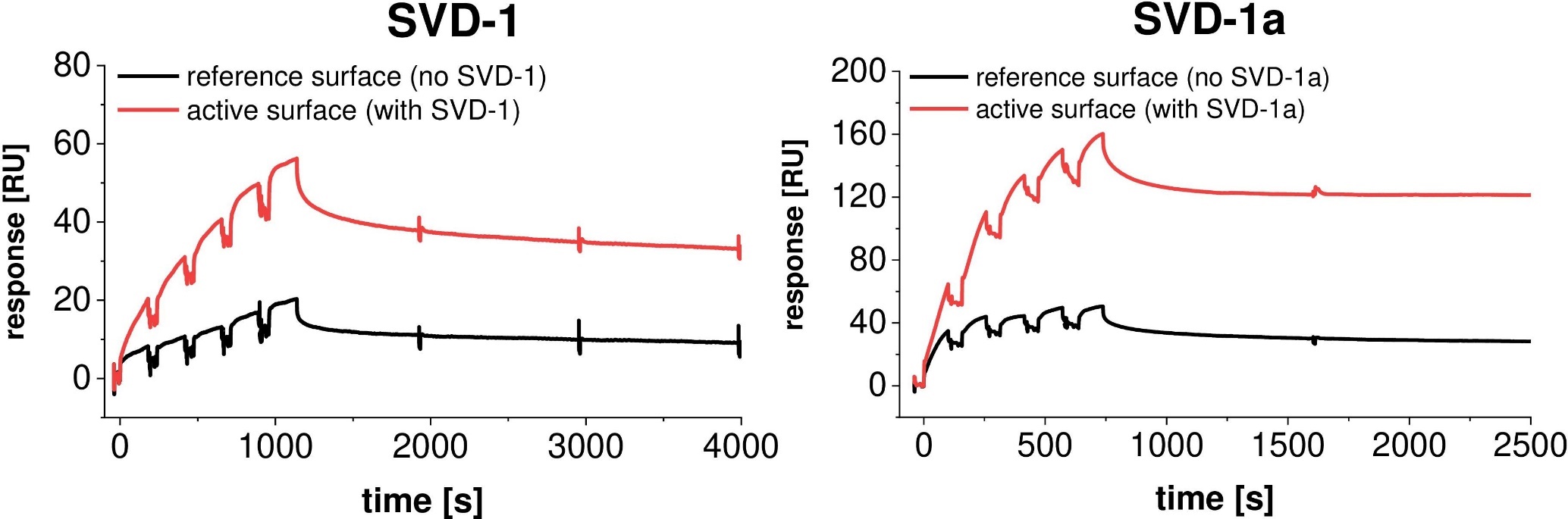


**Supplementary Figure 7: Single cycle injections of α-syn on active surface with immobilized SVD-1 and SVD-1a and the non-immobilized reference surface**. SVD-1 (left) and SVD-1a (right) were immobilized on a carboxyldextran matrix via amino coupling until saturation was reached (CMD200M, Xantec, GE). Full-length a-syn was injected for 100 s for each concentration at 30 µl/min in PBS 7.4 on the active sensor with immobilized SVD peptide (red) and on the empty reference surface (black). Injections were performed using a serial dilution in the range of 30 to 500 nM and 30 to 150 nM for SVD-1 and SVD-1a, respectively. Referenced data are shown in Fig. 7.


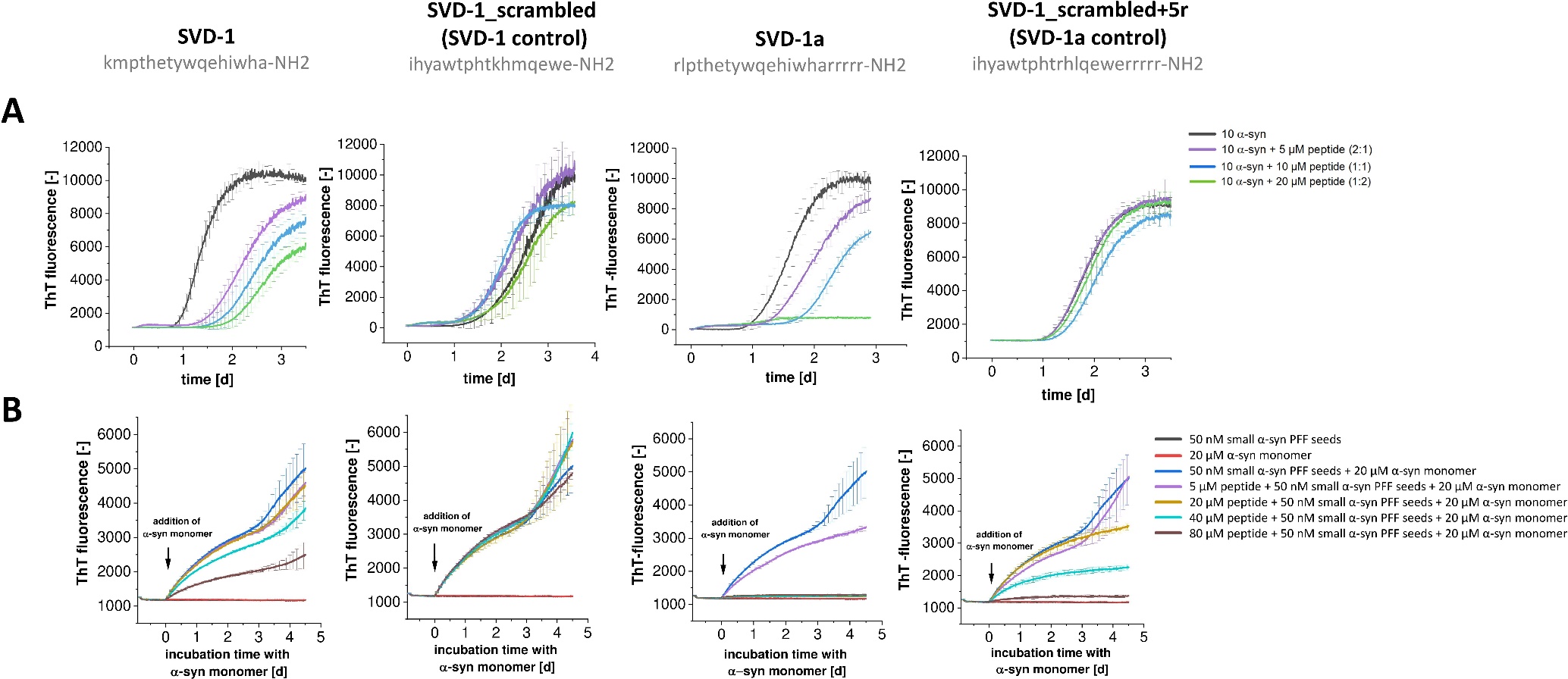


**Supplementary Figure 8: De novo and seeded ThT aggregation with SVD-1 and SVD-1a and the respective randomized control peptides (SVD-1_scrambled and SVD-1_scrambled+5r)**. ThT fluorescence progression was measured in a 96-well non-binding half-area plate (Corning, USA) with a FLUOStar platereader (BMG labtech, GE) at λex = 448 nm and λem = 482 nm. D-peptide sequences are shown as single letter amino acid code in grey. (**A**) *De novo* aggregation assay. 10 µM α-syn monomer was incubated with 5, 10 and 20 µM peptide at 37 °C, adding one borosilicate glass bead per well (d = 3.0 mm, Hilgenberg, GE) with continuous orbital shaking at 300 rpm in between reads. Mean data shown with ± SD (n = 5). The statistical evaluation on significance of inhibitory effects is shown in Supplementary Fig. 9. Please note, when comparing the lag-times in this figure with those in Supplementary Figures 4 to 6, that shaking conditions were different. (**B**) Seeded aggregation assay. 50 nM monomer equivalent small α-syn PFF as seeds were pre-incubated for 20 h with or without peptide at 37 °C under quiescent conditions. Only then, 20 µM α-syn monomer was added for induction of seeding and the incubation time with α-syn monomers started*.* Mean data shown with ± SD (n = 3).


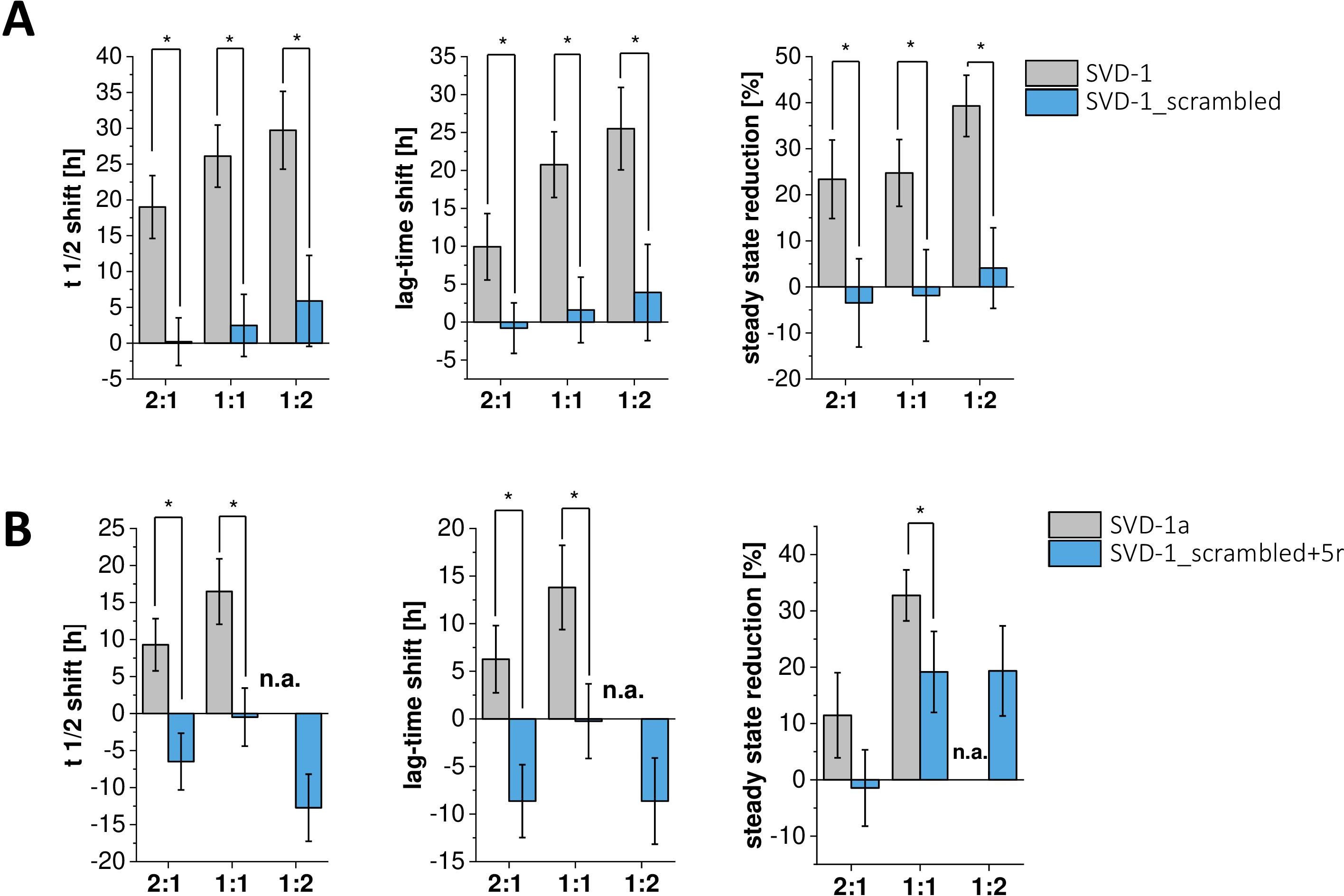


**Supplementary Figure 9: Statistical evaluation of *de novo* aggregation t ½-, lag-time shifts as well as steady state reduction for SVD-1 and SVD-1_scrambled as shown in Supplementary Fig. 8** The five replicates of each condition were individually fitted with a symmetric Boltzmann fit. For each fit, x ½, lag-time and steady state response was determined in order to calculate their mean value for each condition. The t ½ is given by the inflection point of the fit and the lag-time was approximated using the formula [lag-time = t ½ - 2dx] where dx is defined as the slope of the fit at x = t ½. t ½ and lag-time shifts were determined based on the differences between samples with and without peptide, whereas the 0-value represents the reference control without inhibitor. The 2:1 stoichiometric sample of SVD-1a was not fitted due to lacking signal intensity and thus is marked with n.a.. α-Syn untreated control aggregation time: SVD-1: t ½ 32.9 h, lag-time 23.0 h; SVD-1_scrambled: 43.0 h; lag-time: 31.2 h; SVD-1a: t ½ 38.2 h; lag-time 27.2 h; SVD-1_scrambled+5r: t ½ 60.8 h; lag-time: 44.5 h. The significance of time-shifts between sample and the same concentration of the according control peptide was tested with a two sample Welch‘s t-test with p < 0.05 (significant: *). Mean data shown with ± SD (n = 5).


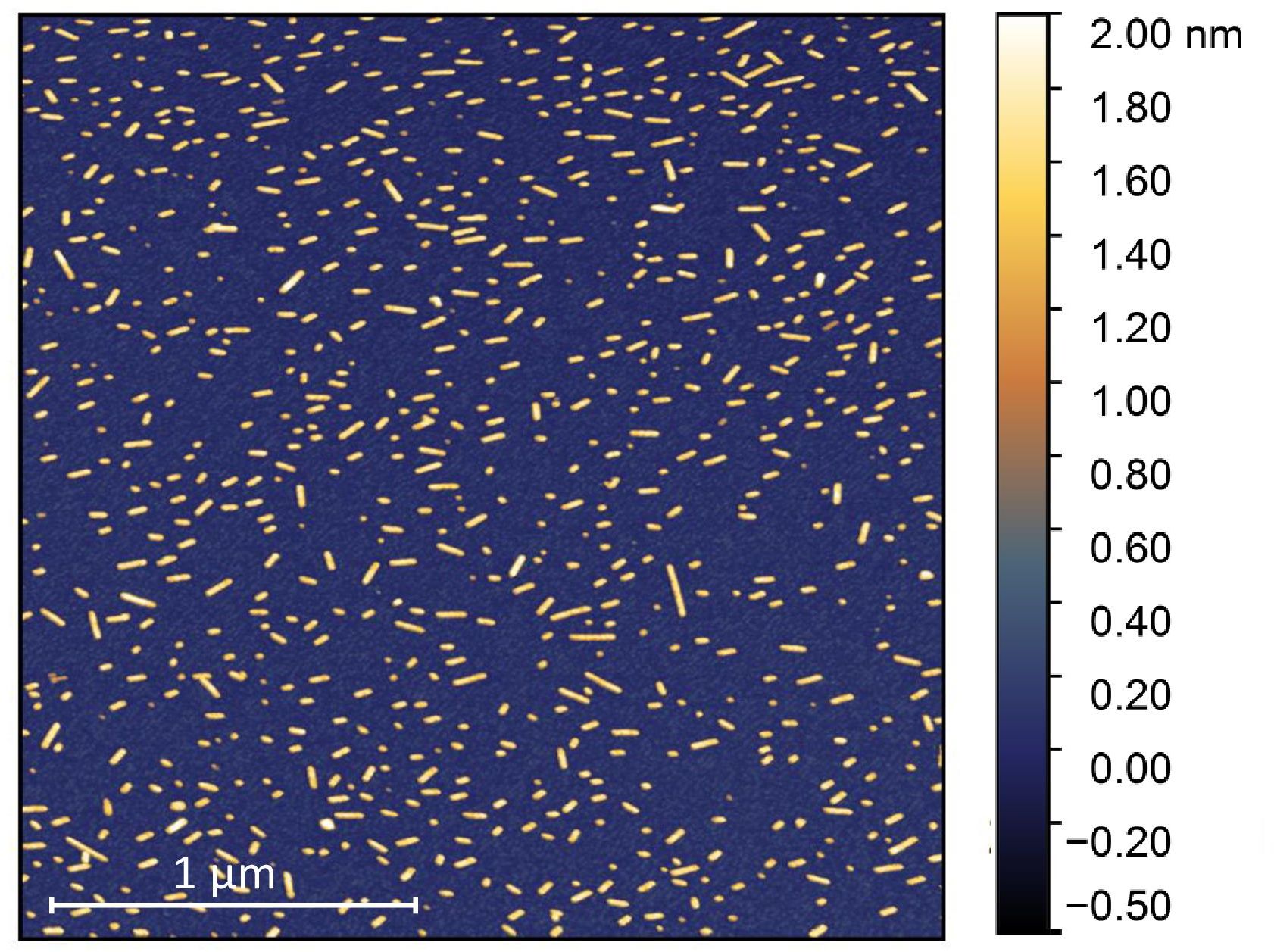


**Supplementary Figure 10: Atomic force microscopy image of small α-syn PFF seed preparations.** The preparation after ultracentrifugation was diluted to a final concentration of 5 µM monomer equivalent in PBS pH 7.4. 5 µl was incubated for 30 min at RT on a freshly cleaved mica, washed three times with distilled water and dried in a gentle stream of N_2_. Measurements were performed in a Nanowizard 3 system (JPK Instruments AG, GE) using intermittent contact mode with 2.5 x 2.5 µm section and line rates of 0.5–1 Hz in ambient conditions with a silicon cantilever with nominal spring constant of 26 newtons/m and average tip radius of 7 nm (Olympus OMCL-AC160TS).


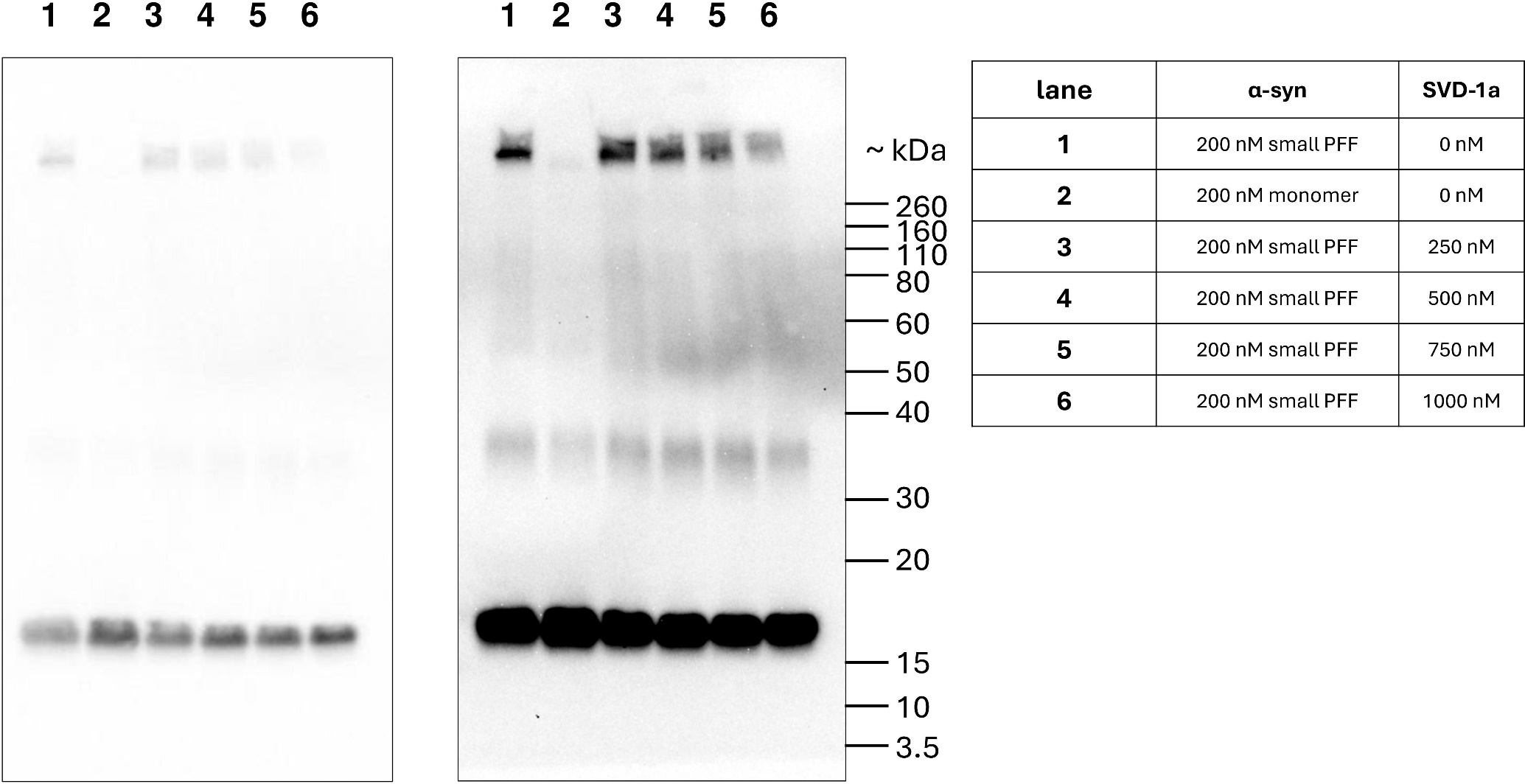


**Supplementary Figure 11**: **Western blot of small α-syn PFF samples incubated with SVD-1a.** 200 nM small α-syn PFFs (monomer equivalent) were incubated with increasing concentrations of SVD-1a for 72 h at 37 °C. Image of whole lanes (left: short exposure, right: long exposure); contrast settings were adjusted for better visibility on screen. A PFF and monomer sample that were incubated without SVD-1a served as controls. α-Syn was detected with Syn211. A prominent monomer band was present between the 15 kDa- and 20 kDa marker bands, detected with shorter exposure time (left). Below the monomer bands, no further bands were visible. In the upper part of the blot, above the marker range, PFFs were detected using longer exposure time due to weaker signal (right).


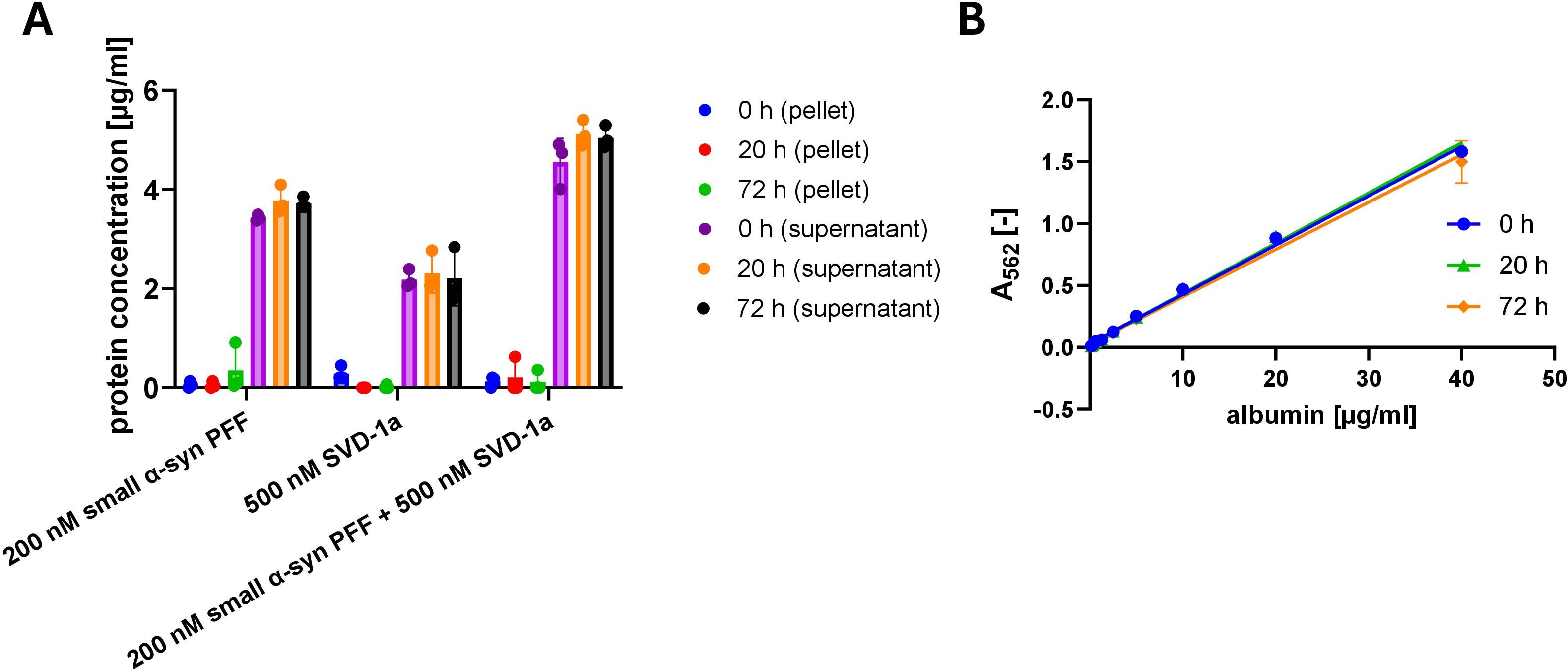


**Supplementary Figure 12: The incubation product of SVD-1a and small α-syn PFF** **is soluble**. (**A**) Samples were prepared as shown in Fig. 4 (A) together with an SVD-1a control and incubated at 37 °C under quiescent conditions in low binding reaction tubes (Eppendorf, GE). After 0, 20 and 72 h samples were centrifuged at 21.000 xg for 30 min at RT. The supernatants were transferred to fresh reaction tubes and any remaining pellets were resuspended (n = 3). (**B)** Albumin calibration with concentrations of 0.15 – 40 µg/ml (n = 2).


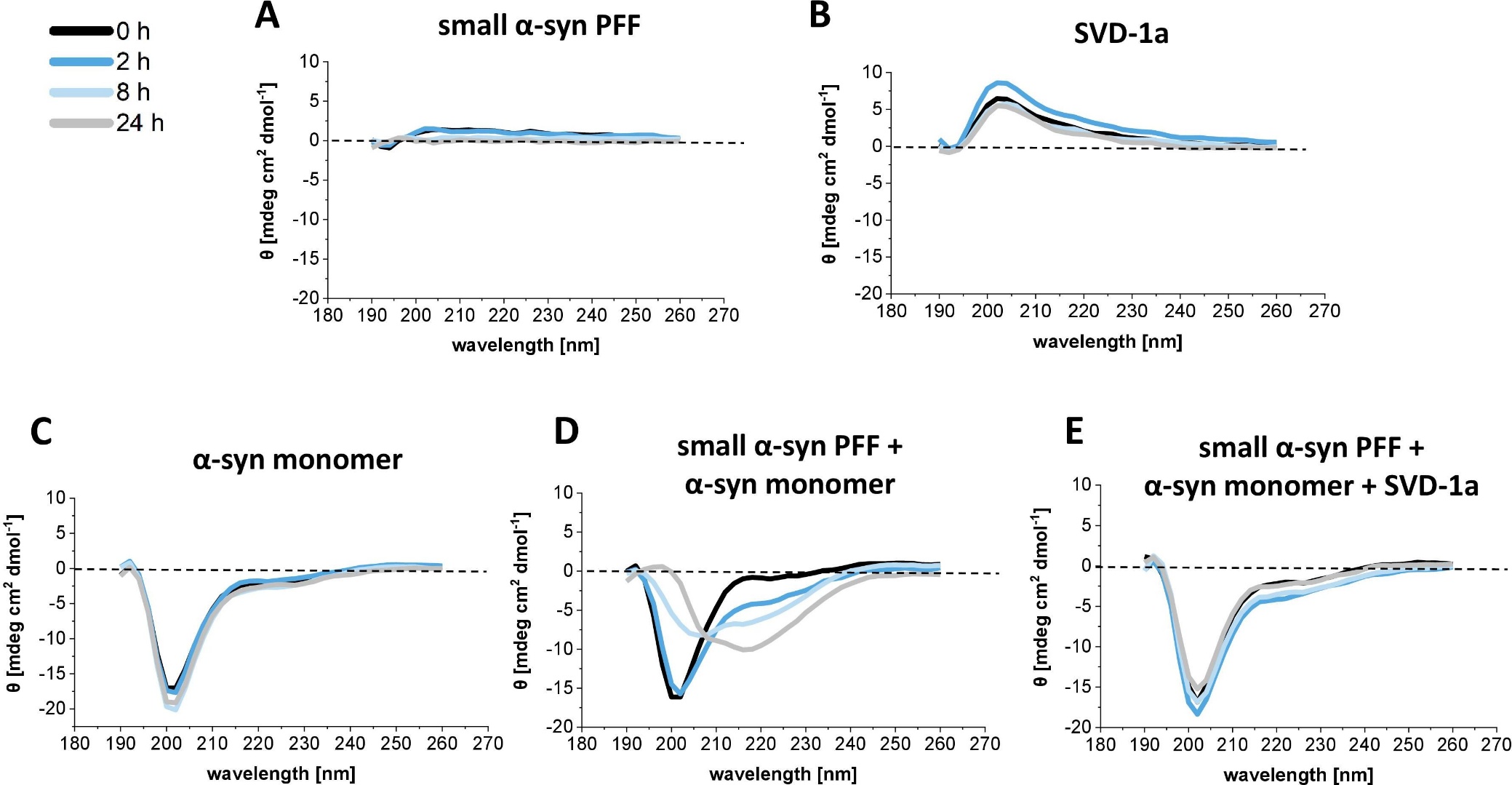


**Supplementary Fig. 13: Analysis of the seeded aggregation assay in the presence and absence of SVD-1a using circular dichroism (CD) spectroscopy**. Small α-syn PFF (500 nM) were incubated in PBS (pH 7.4) at 37 °C under quiescent conditions for 24 hours, either alone or in the presence of 15 µM α-synuclein monomer and/or 30 µM SVD-1a. For each condition and time point, three replicates (each 120 µl) were pooled and analyzed by CD spectroscopy at 0, 2, 8, and 24 hours. Far-UV ellipticity was measured in a quartz cuvette (path length = 10 mm) using a J-1100 CD spectrometer (Jasco, GE). (**A**) Incubation of 500 nM α-synuclein PFFs alone did not yield detectable CD spectra due to their low concentration. (**B**) SVD-1a alone at 30 µM exhibited a CD spectrum characteristic of a random coil secondary structure, consistent with the expected conformation of D-enantiomeric peptides. (**C**) Incubation of 15 µM α-synuclein monomer in the absence of PFFs did not result in any secondary structure transition, as evidenced by a persistent random coil spectrum across all time points. (**D**) Co-incubation of 500 nM α-synuclein PFFs with 15 µM α-synuclein monomer induced a structural transition towards β-sheet formation, as reflected in the CD spectrum (referenced to spectrum in (A)). (**E**) The addition of 30 µM SVD-1a to the condition described in (D) stabilized the α-synuclein monomer in its random coil conformation throughout the incubation period (spectrum referenced to (A) and (B)).


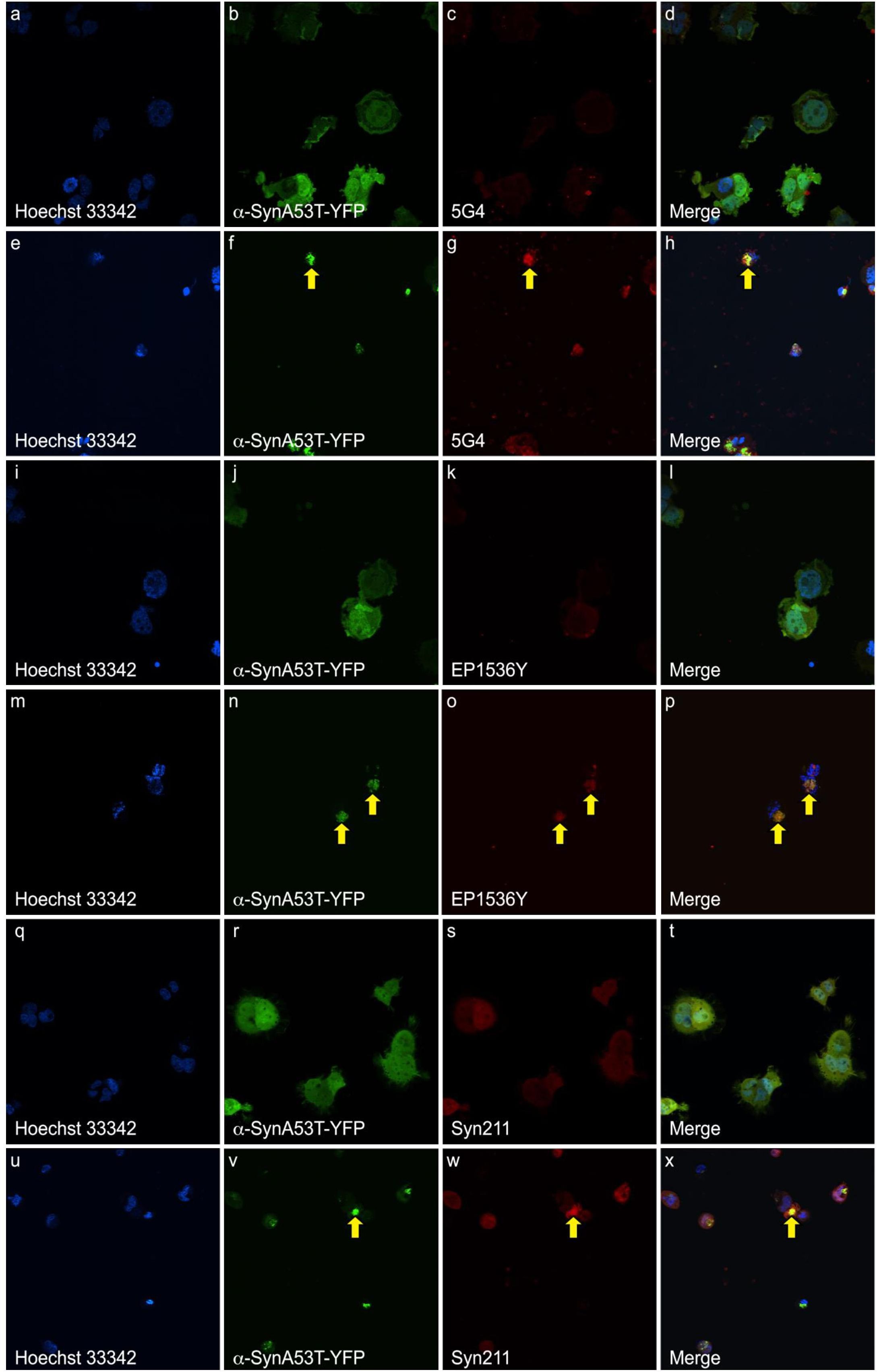


**Supplementary Figure 14: Immunofluorescence staining of α-syn aggregates in α-synA53T–YFP cells.** After culturing unseeded cells (a–d, i–l, and q–t) and cells seeded with PFF (e–h, m–p, and u–x) for 3 days, cells were fixed, permeabilized, and stained with fluorescently labeled antibodies (red). For detecting oligomeric and fibrillar α-syn, we used the anti-aggregated α-syn antibody, clone 5G4 (c, d, g, h). For detecting α-syn phosphorylated at serine 129, which accumulates when α-syn aggregates and forms deposits, we used the recombinant anti-α-syn (phospho S129) antibody EP1536Y (k, l, o, p). For detecting total αsyn, we used the anti-α-syn antibody syn211 (s, t, w, x). Nuclei were stained with Hoechst 33342. α-synA53T–YFP fluorescence is shown in green. Panels on the right represent merged images (d, h, l, p, t, x). While α-synA53T–YFP fluorescence is diffusely distributed in unseeded cells, it forms foci in cells seeded with PFF, indicative of α-syn aggregation. Yellow arrows indicate aggregated α-syn (yellow), a colocalization of antibody staining for α-syn (red) and fluorescence of aggregated α-synA53T–YFP (green). The scale bar indicates 100 µm.


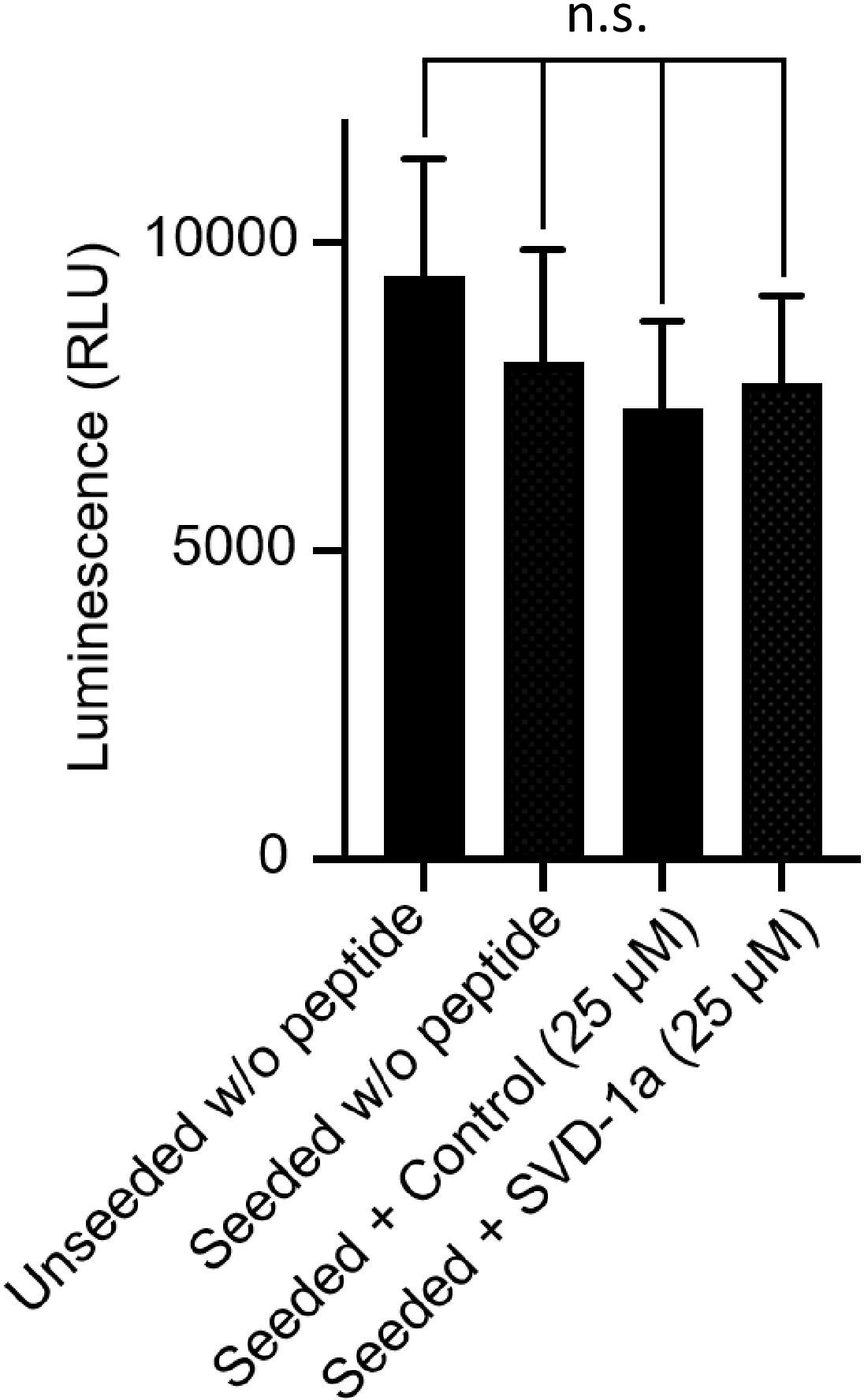


**Supplementary Figure 15: The transfection of small α-syn PFF** **and SVD-1a does not significantly reduce HEK293 cell viability.** CellTiter-Glo Luminescent Cell Viability Assay (Promega GmbH, GE) was used to determine the number of viable cells in culture based on quantitation of the ATP present, an indicator of metabolically active cells. After culturing cells in 384-well plates for three days, 35 µl of medium was removed from the wells and 40 µl of CellTiter-Glo Reagent directly added to each well. After mixing, luminescence was measured 10 min later using a Fluostar (BMG labtech, GE). The luminescence of seeded αSynA53T–YFP cells was slightly lower than that of unseeded cells, however, the difference was not significant. Also, treatment with a negative-control peptide or with SVD-1a did not induce significantly different luminescence in comparison to unseeded cells or seeded cells without peptide treatment. Significance was calculated using one-way ANOVA followed by Dunnett's multiple comparisons test with p < 0.05.


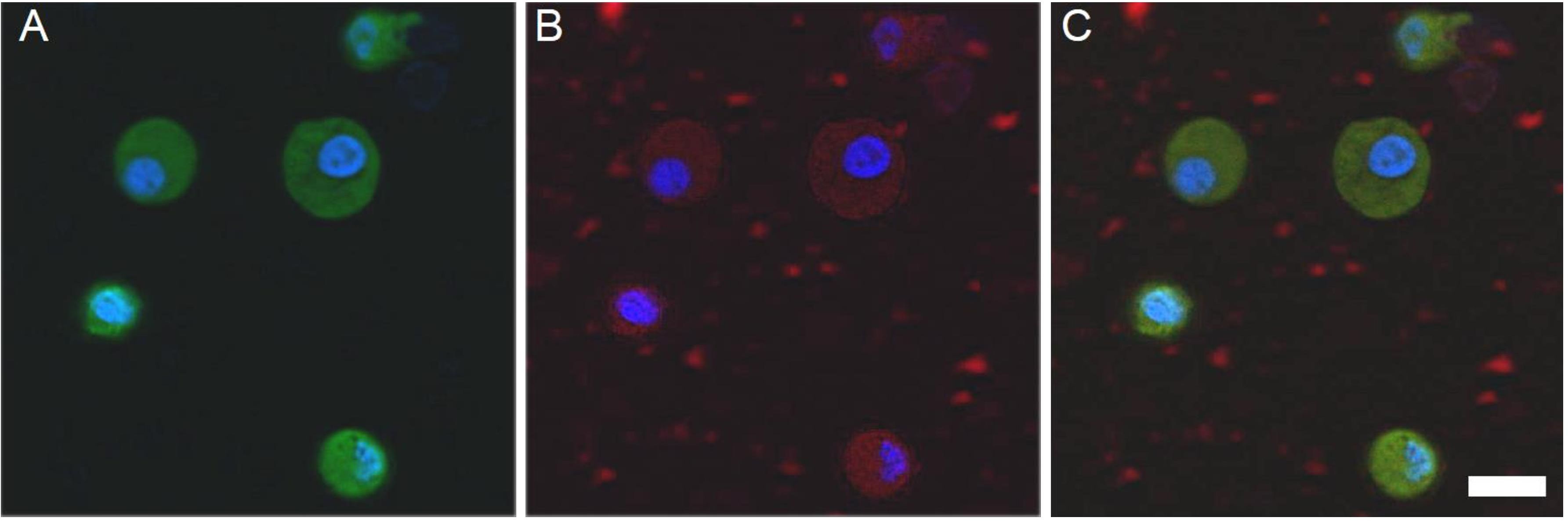


**Supplementary Figure 16: Fluorescently labeled SVD-1aCys_Alexa647 is taken up in α-synA53T–YFP cells**. Unseeded α-synA53T–YFP cells were supplemented with 5 µM of SVD-1aCys_Alexa647 in the cell culture medium for 24 h. Confocal images of the cells show a diffuse green signal for cytoplasmic α-synA53T–YFP (**A**), and a diffuse red signal for SVD-1aCys_Alexa647 that was labeled with a red fluorescent dye and taken up by the cells (**B**). Nuclei were stained with Hoechst 33342 in blue. The last panel represents a merged image (**C**). The scale bar indicates 20 µm. Because the red fluorophore signal was homogeneously distributed in the cytosol of the cells, we assume that the peptide is able to enter the cells even without transfection.


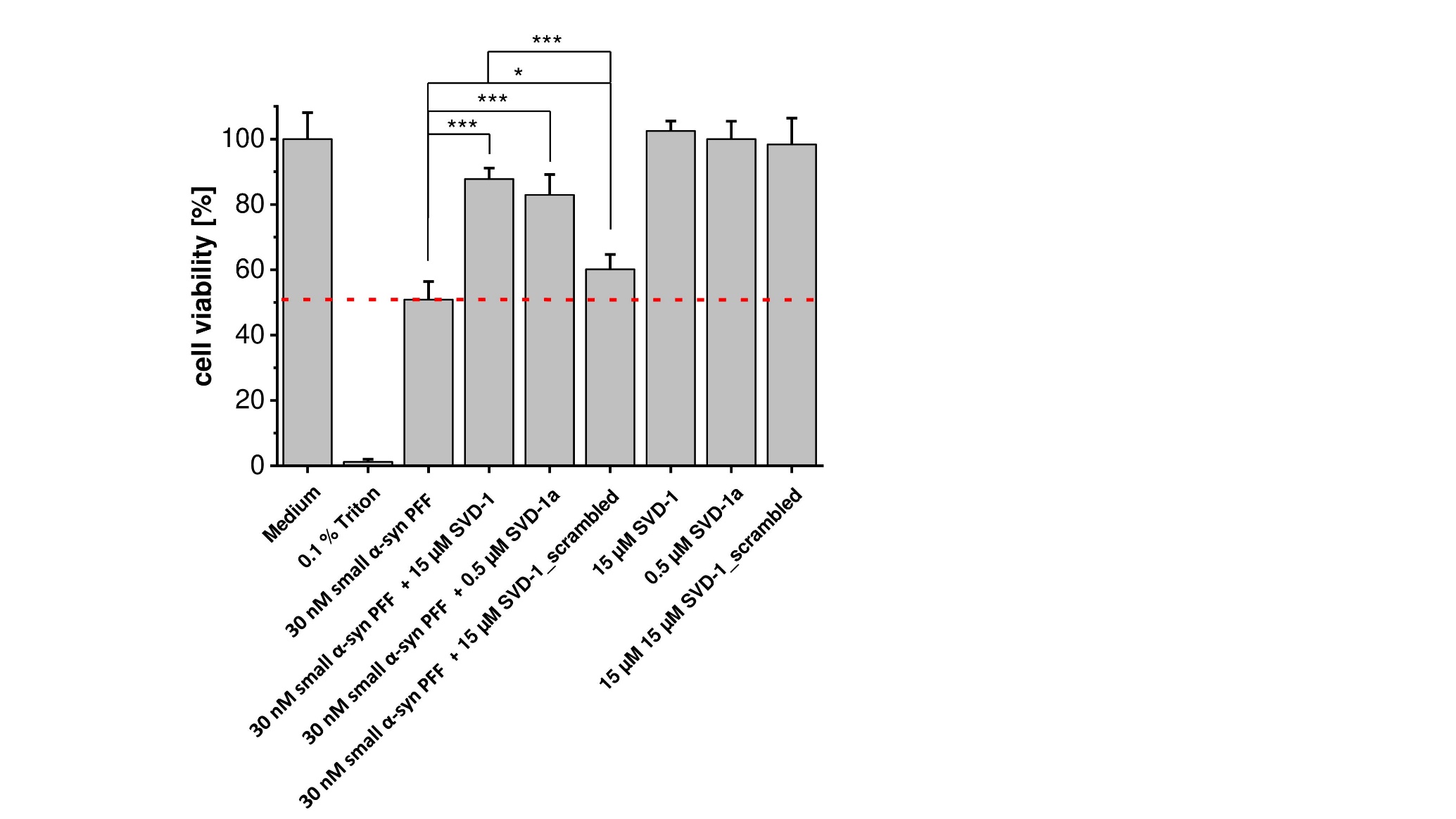


**Supplementary Figure 17: SVD-1 and SVD-1a increase cell viability in MTT assay with small α-syn PFF** **seeds.**  SVD-1, SVD‑1a SVD-1_scrambled were incubated over night at 37 °C and 300 rpm with or without 30 nM PFF oligomers and subsequently incubated with PC12 cells in final concentrations of 15 µM (SVD-1 and SVD-1_scrambled) or 0.5 µM (SVD-1a). While the cell viability was reduced with 30 nM PFF oligomers alone to 50 %, 89 and 82 % of cells were rescued in the presence of SVD-1 and SVD-1a, respectively. Slight increase of cell viability saving effect was also detected for 15 µM of the control peptide SVD-1_scrambled. Test on significance was performed using one-way ANOVA with Bonferroni post hoc analysis: * p ≤ 0.5, *** p ≤ 0.01. Mean data are shown with ±SD (n = 4). While the cell viability was reduced by 50 % in the presence of 30 nM soluble small α-syn PFF alone, no toxic effect of the compounds on PC12 was observed. However, when the seeds were pre-incubated with SVD-1 or SVD-1a, the cell viability increased to 89 and 82 % using 15 µM or 0.5 µM compound concentrations, respectively. Thus, similar cell viability saving effects were found for SVD-1a using a 30-fold lower concentration. For the concentration of 15 µM SVD 1_scrambled, only a slight cell viability saving effect was found which was significantly lower than the cell saving effect identified for the same concentration of SVD‑1. This result shows that the efficacy of the compounds on the soluble α-syn PFF is not only limited to a seeding capacity reduction, but additionally, the compounds also reduce the toxicity of the seeds in the cellular environment.


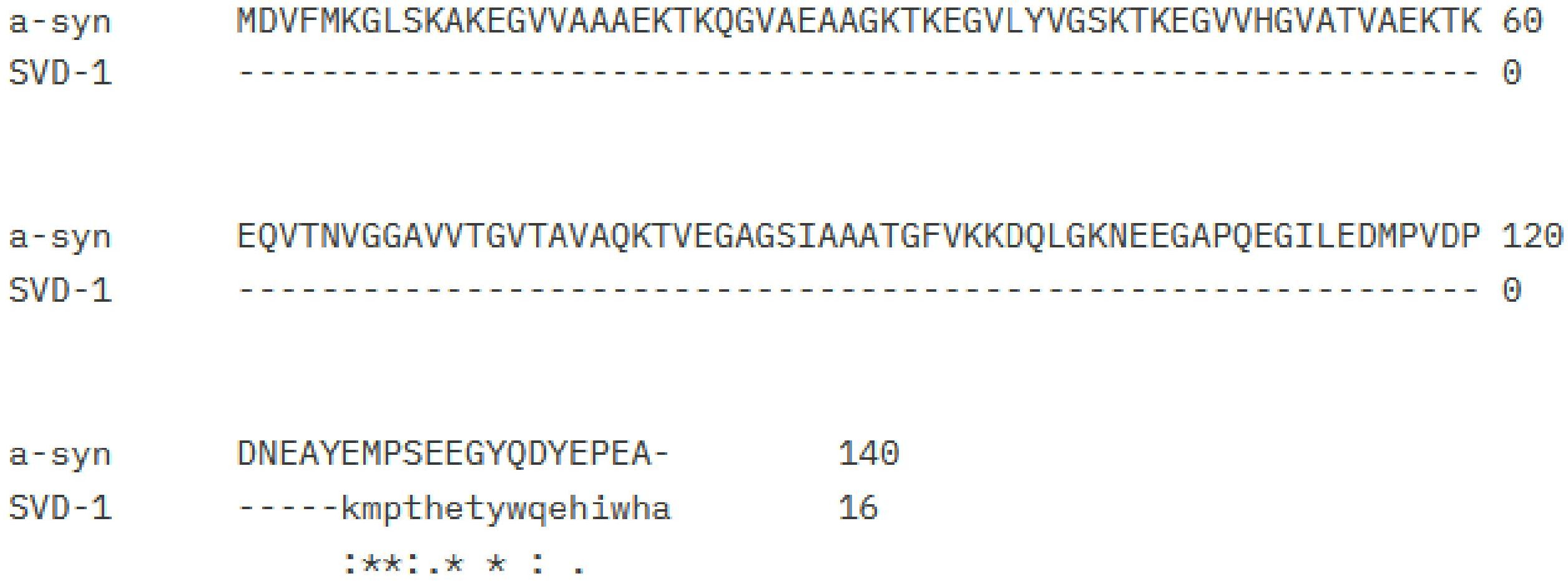


**Supplementary Figure 18: Sequence alignment of human α-syn WT sequence and SVD-1.** Multiple sequence alignment (MSA) was performed using the Clustal Omega application provided by EMBL-EBI. Positions marked with asterisks indicate identical positions, while positions marked with one or two dots indicate medium or high similarity concerning the overall side chain properties, respectively.


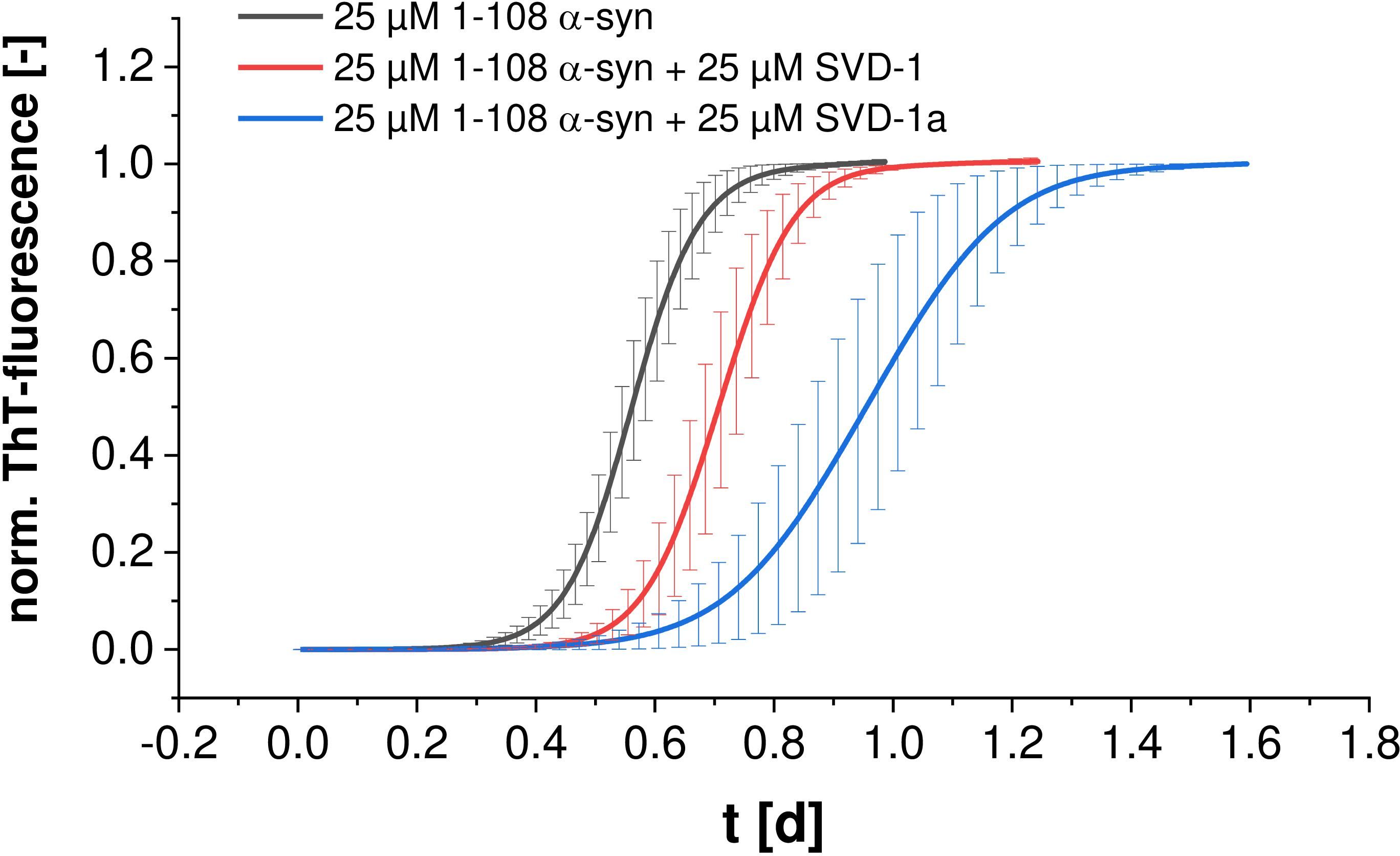


**Supplementary Figure 19: SVD-1 and SVD-1a inhibit the aggregation of 1-108 truncated α-syn.** 25 µM monomeric 1-108 α syn was incubated with or without equimolar concentrations of SVD-1 and SVD-1a as described in the method section for *de novo* ThT-aggregation screening set-up. Aggregation curves were fitted with a symmetric Boltzmann fit and the steady state signal was normalized to 1. SVD-1 as well as SVD-1a show delay of 1- 108 truncated α-syn aggregation.


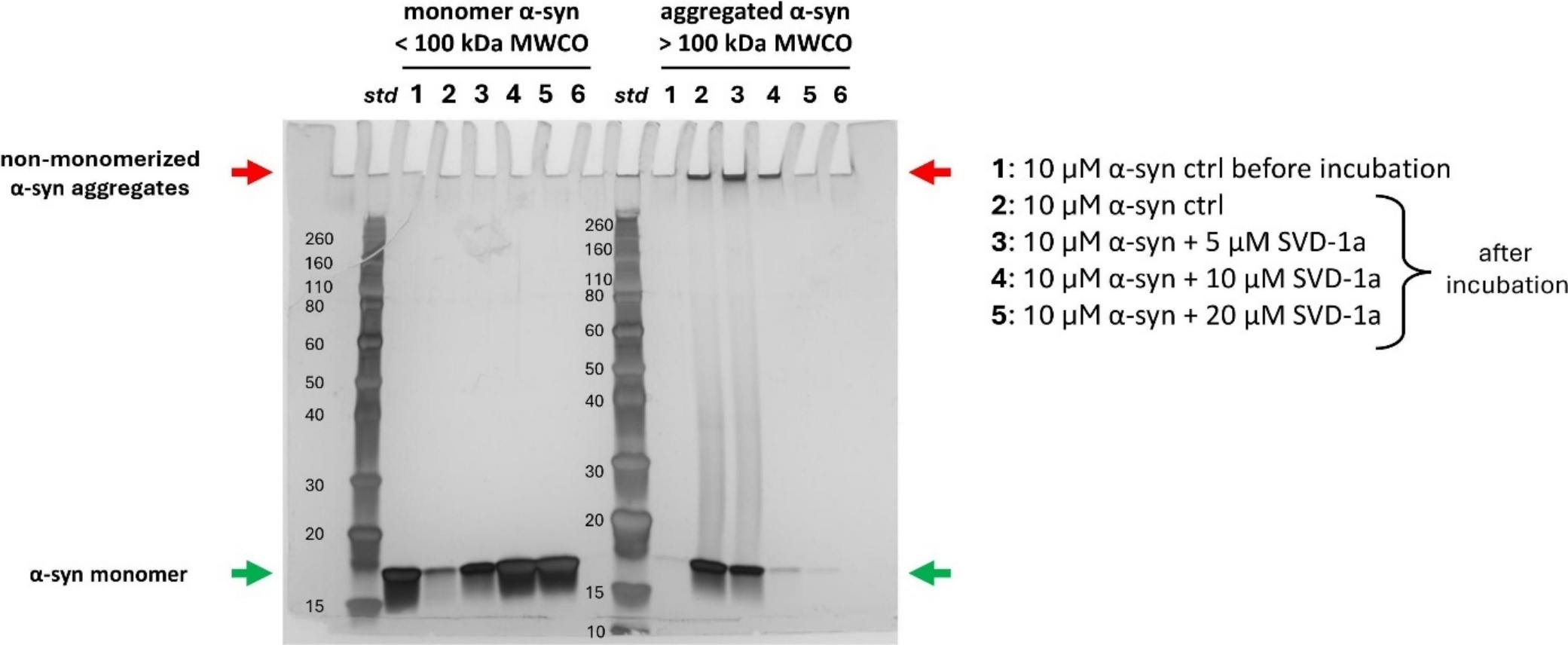


**Supplementary Fig. 20: SDS-PAGE analysis of de novo aggregation samples at incubation endpoint in presence or absence of SVD-1a.** 10 µM α-syn monomer was incubated with or without 5, 10 or 20 µM SVD-1a for 7d in a 96-well non-binding half-area plate (Corning, USA) in a FLUOStar platereader (BMG labtech, GE) with 300 rpm continuous orbital shaking in between reads adding one borosilicate bead per well (d = 3 mm, Hilgenberg, GE, n = 7). The replicates were then united and loaded onto a 100 kDa MWCO centrifugal concentrator. In addition, a 10 µM α-syn sample was prepared and directly subjected to size-based fractionation without being pre-incubated. Retentate and flow through samples were analyzed using SDS-PAGE with silver staining. In the absence of pre-incubation, the majority of α-syn is detected in the retentate (lane 1, left), indicating that it remains predominantly in its monomeric form. Following a 7-day incubation under aggregating conditions, α-syn is almost entirely retained in the retentate (lane 2, right), consistent with extensive aggregation. Upon addition of increasing concentrations of SVD-1a, a progressive shift of α-syn from the retentate to the flow-through is observed (lanes 3–5, left), indicating a concentration-dependent inhibition of aggregation and preservation of the monomeric state. Correspondingly, the amount of α-syn in the retentate decreases with increasing SVD-1a concentrations (lanes 3–5, right). Notably, a fraction of the aggregated α-syn resists denaturation by SDS and boiling and therefore accumulates near the loading pocket in the gel (red arrows), representing non-monomerized α-syn aggregates. However, monomeric α-syn is observed at the expected size (green arrows).
